# Lesions hyper- to isointense to surrounding liver in the hepatobiliary phase of gadoxetic acid-enhanced MRI

**DOI:** 10.1007/s00330-024-10829-x

**Published:** 2024-06-20

**Authors:** Alicia Furumaya, François E. J. A. Willemssen, Razvan L. Miclea, Martijn P. D. Haring, Robbert J. de Haas, Shirin Feshtali, Inge J. S. Vanhooymissen, Daniel Bos, Robert A. de Man, Jan N. M. Ijzermans, Joris I. Erdmann, Joanne Verheij, Michail C. Doukas, Otto M. van Delden, Maarten G. J. Thomeer

**Affiliations:** 1 Department of Surgery, Amsterdam UMC, University of Amsterdam, Amsterdam, The Netherlands; 2 Cancer Center Amsterdam, Amsterdam, The Netherlands; 3 Department of Radiology and Nuclear Medicine, Erasmus MC, Erasmus University Rotterdam, Rotterdam, The Netherlands; 4 Department of Radiology and Nuclear Medicine, Maastricht University Medical Center+, Maastricht University, Maastricht, The Netherlands; 5 Department of Surgery, University Medical Center Groningen, University of Groningen, Groningen, The Netherlands; 6 Department of Radiology, University Medical Center Groningen, University of Groningen, Groningen, The Netherlands; 7 Department of Radiology, Leiden UMC, Leiden University, Leiden, The Netherlands; 8 Department of Radiology, Amsterdam UMC, Vrije Universiteit Amsterdam, Amsterdam, The Netherlands; 9 Department of Epidemiology, Erasmus MC, Erasmus University Rotterdam, Rotterdam, The Netherlands; 10 Department of Gastroenterology and Hepatology, Erasmus MC, Erasmus University Medical Center, Rotterdam, The Netherlands; 11 Department of Surgery, Erasmus MC, Erasmus University Medical Center, Rotterdam, The Netherlands; 12 Department of Pathology, Amsterdam UMC, University of Amsterdam, Amsterdam, The Netherlands; 13 Department of Pathology, Erasmus MC, Erasmus University Medical Center, Rotterdam, The Netherlands; 14 Department of Radiology, Amsterdam UMC, University of Amsterdam, Amsterdam, The Netherlands

**Keywords:** Magnetic resonance imaging, Liver cell adenoma, Hepatocellular carcinoma, Focal nodular hyperplasia, Gadolinium DTPA

## Abstract

**Objectives:**

Hyper- or isointensity in the hepatobiliary phase (HBP) of gadoxetic acid-enhanced MRI has high specificity for focal nodular hyperplasia (FNH) but may be present in hepatocellular adenoma and carcinoma (HCA/HCC). This study aimed to identify imaging characteristics differentiating FNH and HCA/HCC.

**Materials and methods:**

This multicenter retrospective cohort study included patients with pathology-proven FNH or HCA/HCC, hyper-/isointense in the HBP of gadoxetic acid-enhanced MRI between 2010 and 2020. Diagnostic performance of imaging characteristics for the differentiation between FNH and HCA/HCC were reported. Univariable analyses, multivariable logistic regression analyses, and classification and regression tree (CART) analyses were conducted. Sensitivity analyses evaluated imaging characteristics of B-catenin-activated HCA.

**Results:**

In total, 124 patients (mean age 40 years, standard deviation 10 years, 108 female) with 128 hyper-/isointense lesions were included. Pathology diagnoses were FNH and HCA/HCC in 64 lesions (50%) and HCA/HCC in 64 lesions (50%). Imaging characteristics observed exclusively in HCA/HCC were raster and atoll fingerprint patterns in the HBP, sinusoidal dilatation on T2-w, hemosiderin, T1-w in-phase hyperintensity, venous washout, and nodule-in-nodule partification in the HBP and T2-w. Multivariable logistic regression and CART additionally found a T2-w scar indicating FNH, less than 50% fat, and a spherical contour indicating HCA/HCC. In our selected cohort, 14/48 (29%) of HCA were B-catenin activated, most (13/14) showed extensive hyper-/isointensity, and some had a T2-w scar (4/14, 29%).

**Conclusion:**

If the aforementioned characteristics typical for HCA/HCC are encountered in lesions extensively hyper- to isointense, further investigation may be warranted to exclude B-catenin-activated HCA.

**Clinical relevance:**

Hyper- or isointensity in the HBP of gadoxetic acid-enhanced MRI is specific for FNH, but HCA/HCC can also exhibit this feature. Therefore, we described imaging patterns to differentiate these entities.

**Key Points:**

- *FNH and HCA/HCC have similar HBP intensities but have different malignant potentials*.
- *Six imaging patterns exclusive to HCA/HCC were identified in this lesion population*.
- *These features in liver lesions hyper- to isointense in the HBP warrant further evaluation*.

## Introduction

Gadoxetic acid (Gd-EOB-DTPA) enhanced magnetic resonance imaging (MRI) is increasingly applied for several indications, including detection of premalignant and secondary liver lesions, characterization of liver lesions, and assessment of liver (dys)function [1, 2]. Clinically relevant primary liver lesions include focal nodular hyperplasia (FNH), hepatocellular adenoma (HCA), and hepatocellular carcinoma (HCC) [3].

FNH mostly occurs in female patients between 35 years and 50 years, has no risk of malignant transformation or bleeding, and does not require follow-up [4]. HCA also mostly occurs in young women. HCA does have a risk of malignant transformation (± 5%) and bleeding (15–20%). Therefore, HCA may require further follow-up or treatment, depending on their molecular subtype, size, location, and patient’s sex [4–6]. HCC is the malignant counterpart of HCA, mostly seen in men between 70 years and 79 years. Treatment is recommended according to the European Association for the Study of the Liver (EASL) guidelines [7].

Adequate differentiation between FNH and HCA/HCC has clinical consequences. Overdiagnosis of FNH as HCA/HCC can lead to unnecessary diagnostic procedures (including biopsy) and/or treatment with potential complications. Conversely, underdiagnosing HCA/HCC as FNH can result in inadequate treatment and thus to malignant transformation and/or metastasis. Clinical characteristics such as sex and underlying liver disease are helpful but do not fully discriminate, and there are no biomarkers to differentiate FNH and HCA/HCC.

According to current EASL guidelines, hyper- or isointensity in the hepatobiliary phase (HBP) of Gd-EOB-DTPA enhanced MRI has high sensitivity and specificity for FNH, whereas HCA/HCC is generally characterized by hypointensity in the HBP [4, 8, 9]. However, more than 80% of B-catenin-activated HCA, the molecular subtype with the highest risk of malignant transformation, are hyper- or isointense in the HBP [1, 10–13]. Hyper- or isointensity is also seen in approximately 10% of HCC [1, 10, 14–16].

Quantitative analysis of the extent of hyper- or isointensity has been suggested to improve the differentiation between lesions hyper- or isointense in the HBP [17–19]. However, quantitative HBP analysis may be hampered by heterogeneity, often caused by imaging patterns within the lesions [20, 21]. For example, the inflammatory HCA subtype is characterized by the “atoll sign”, illustrated by a hyperintense peripheral rim on T2-weighted images [22, 23].

Data combining evidence on hyper- or isointensity in the HBP with MR imaging patterns are limited [24]. Therefore, the aim of the current study was to identify imaging characteristics to aid the differentiation between FNH and HCA/HCC that were hyper- or isointense in the HBP on Gd-EOB-DPTA enhanced MRI.

## Materials and methods

### Study design and inclusion in participating center

A retrospective cohort study was performed in accordance with the Declaration of Helsinki and the STROBE guidelines. The need for ethical approval was waived. If required by regulations of participating centers, patients not occurring in objection registers were sent a letter detailing study procedures and aims and could opt out of the study. All patients with pathology-proven FNH, HCA, or HCC (biopsies or resection specimens) between 2010 and 2020 were identified through local pathology databases. Radiologists in participating centers reviewed all Gd-EOB-DTPA-enhanced MRIs. All lesions hyper- or isointense compared to the surrounding liver in the HBP by visual interpretation were included, including partially hyper- to isointense lesions.

### Central inclusion and imaging techniques

All hyper- or isointense lesions were centrally pseudonymously reviewed by two expert radiologists (more than twenty years of experience) blinded from the pathological classification. Included were patients with hyper- or isointense regions in the lesions compared to the surrounding liver, not attributable to pre-contrast signal intensity, therefore due to contrast uptake in the HBP. In case of doubt, subtraction images were produced.

In general, MRI scans were conducted using 1.5-Tesla or 3.0-Tesla scanners. Typically, a spoiled 3D gradient fat-saturated T1-weighted sequence was produced before and during the different dynamic phases after contrast injection. The HBP consisted of a similar sequence as the dynamic sequence, and was performed between 10 min and 20 min after contrast injection. In- and opposed-phase T1-weighted sequence was performed using the Dixon technique, if available. T2-weighted 2D spin-echo sequence was performed with or without fat saturation, using a single shot or/and multishot protocol. Diffusion-weighted sequences included typically minimally two *b*-values with the first between 0 s/mm² and 150 s/mm² and the second from 600 s/mm² to 800 s/mm².

### Imaging characteristics

The supplementary material shows full signs, definitions, and scoring process. Patterns in the HBP were raster, perilesional hypointense rim [25], lesional hyper- or isointense rim [26], atoll fingerprint, white-bordered flower (present/absent), partification (no, craquelure with loosening part, focal/mosaic or nodule-in-nodule) [27, 28], and extent of hyper- or isointensity (limited/extensive). Patterns on T2-w were sinusoidal dilatation (present/absent) [23] and partification (no, focal/mosaic or nodule-in-nodule). Patterns scored in the HBP and T2-weighted images were scar [29], liquid (present/absent), and extent of heterogeneity (no/limited or extensive).

Miscellaneous imaging features were: T1-w in-phase hyperintensity not due to fat, hemosiderin (present/absent), and intralesional fat (absent, less than 50%, or more than 50%). In dynamic phases, intralesional arterial hypointense rim (present/absent) and venous washout (present, absent, equivocal, or pseudo-washout) were scored [9, 30, 31]. If diffusion-weighted imaging (DWI) was available, diffusion restriction was scored (present, absent, or equivocal). Lesion contour was defined as spherical, cauliflower, or aspecific.

Finally, the final diagnosis (i.e., FNH or HCA/HCC) and treatment recommendation (i.e., no follow-up, follow-up, or histology (either biopsy or resection)) were formulated. HCA and HCC were grouped because both are hepatocellular lesions with (risk of) malignant transformation generally assumed to be hypointense in the HBP. Treatment recommendations were based on imaging characteristics alone, not taking lesion size and location into account.

### Pathological and clinical data

Pathological classification was based on a review of pathology reports. The time between MRI imaging and biopsy and/or resection was a median of 0 months (IQR: 0–3 months). The electronic supplementary material describes morphological requirements for each diagnosis. For the differentiation between FNH and HCA, either a pattern of glutamine synthetase (GS) staining or, in the case of HCA, a positive C-reactive protein or positive serum amyloid A staining was required [32]. B-catenin-activated HCA was characterized by nuclear staining of B-catenin and/or overexpression of GS (*n* = 5) and/or molecular evidence of mutations in the *CTNNB1* gene (*n* = 9). If available, the locus of the mutation was noted.

Clinical data and baseline characteristics collected were lesion size in centimeters and lesion location (left or right liver lobe). Pathological and clinical data were collected using RedCap. Underlying liver disease was defined as non-alcoholic fatty liver disease or non-alcohol steatohepatitis, cirrhosis and/or fibrosis, and metabolic diseases including hepatocyte nuclear factor 1A maturity-onset diabetes of the young and glycogen storage disorders.

### Statistical analysis

Statistical analyses were conducted using SPSS^®^ statistics version 28.0 (IBM). Classification and regression tree (CART) analysis was conducted using the recursive partitioning and regression trees (Rpart) package in RStudio version 4.2.1 (Posit). The Rpart package uses ten-fold cross-validation to determine the optimal place to prune the regression tree. Categorical variables were expressed as numerators/denominators with percentages and continuous variables as means with standard deviations (SDs) or medians with interquartile ranges (IQRs). For all analyses, available case analysis was conducted.

Interobserver and intraobserver variability were calculated using Cohen’s kappa statistic. Levels of agreement were interpreted using cut-off values defined by Landis and Koch: < 0.00 poor, 0.00–0.20 slight, 0.21–0.40 fair, 0.41–0.60 moderate, 0.61–0.80 substantial, and 0.81–1.00 almost perfect [33].

In univariable analyses, imaging features were compared between patients with a final pathological diagnosis of FNH and HCA/HCC using Pearson’s chi-squared test or Fisher’s exact test. *p*-values < 0.05 were considered statistically significant. Diagnostic odds ratios with 95% confidence intervals for the diagnosis FNH, compared to HCA/HCC were calculated. Sensitivity, specificity, positive predictive value, and negative predictive value were calculated for significant imaging features. Haldane–Anscombe correction was performed in the case of zero cells.

For multivariable analyses, imaging features with a *p*-value of > 0.25 in univariable analysis were excluded. A Spearman’s correlation matrix of imaging features was derived. In cases of a correlation coefficient of 0.80 and higher, indicative of collinearity, one of the correlated imaging features was excluded from the multivariable analysis. The tree diagram obtained by CART analysis and odds ratios obtained by multivariable logistic regression were combined in a visual representation made in the draw.io® (JGraph Limited). Sensitivity analyses assessed the occurrence of imaging features suggestive of FNH in B-catenin-activated HCA.

## Results

**Fig. 1:**
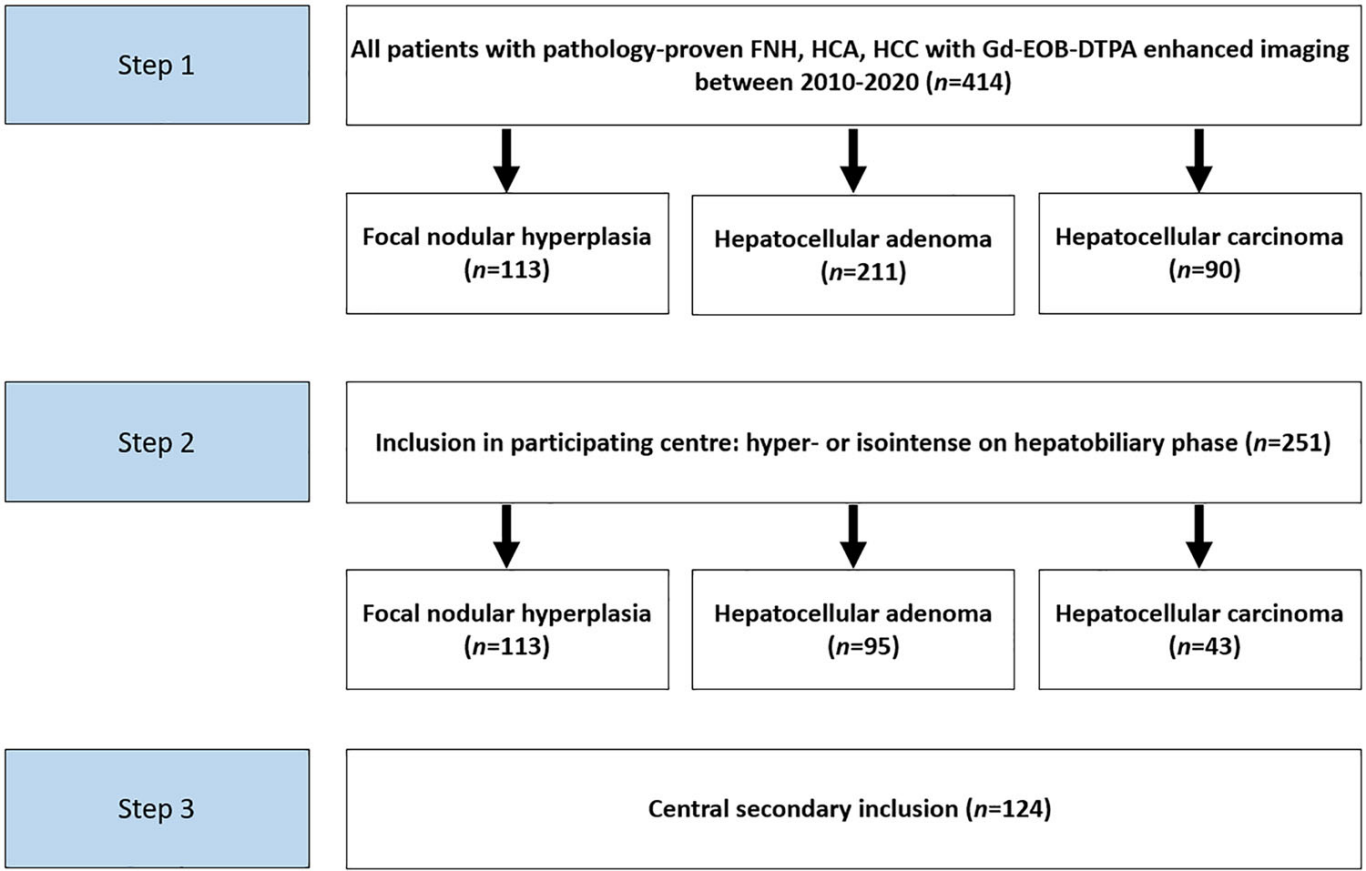
Flowchart of the inclusion process

In the six participating centers, hyper- or isointense lesions were observed in the HBP in 113/113 (100%), 95/211 (45%), and 43/90 (48%) of patients with pathology-proven FNH, HCA, and HCC, respectively. Hyper- or isointense lesions were included for central revision with subtraction images. After central inclusion based on the central assessment of pathological classification and hyper- and isointensity, 124/251 patients (49%) were included for full analysis (Fig. 1).

The majority (108/124, 87%) of patients were female and the mean age was 40.3 years (SD 9.8 years). Underlying liver disease was present in 29/124 patients (23%) and 66/124 patients (53%) had a known history of oral contraceptive use. Two patients (1.6%) had a history of anabolic steroid (ab)use and one patient (0.8%) of transdermal estrogen use (Table 1).

**Table 1:** Baseline characteristics

Clinical parameters	Category	*n* = 124
Age at diagnosis (mean, SD) in years	NA	40.3 (9.8)
Sex (*n*, %)	Female Male	108 (87) 16 (13)
Underlying liver disease (*n*, %)	Yes No	29 (23) 95 (77)
History of hormone use (*n*, %)	Oral contraceptives Other No	66 (53) 3 (2.4) 55 (44)

Lesion parameters	Category	*n* = 128
Lesion size (mean, SD) in centimeters	NA	6.8 (3.3)
Lesion location (*n*, %)	Left Right	71 (55) 57 (45)
Final diagnosis based on imaging	FNH HCA/HCC	66 (52%) 62 (48%)
Treatment recommendations based on imaging	No follow-up Follow-up Biopsy/resection	57 (45%) 12 (9.4%) 59 (46%)
Final diagnosis based on pathology	FNH HCA/HCC	64 (50%) 64 (50%)

### Baseline imaging and pathological characteristics

In total, 124 patients with 128 hyper- or isointense lesions were included. The mean lesion size was 6.8 (SD 3.3) centimeters and most lesions arose from the left 71/128 (55%) hemiliver.

Based on imaging, the 128 lesions were classified as FNH in 66 patients (52%) and HCA/HCC in 62 patients (48%, Table 1). In 12 of the 66 patients (18%) diagnosed with FNH on imaging, biopsy was recommended. Interobserver agreement was moderate and intraobserver agreement was substantial for the majority of the imaging characteristics (Supplementary Tables 1 and 2).

Final pathology diagnoses were evenly distributed between the two groups (FNH in 64 patients, 50%, and HCA/HCC in 64 patients, 50%). Two patients were classified as HCA/HCC on imaging and turned out to be FNH on pathology. Four patients classified as FNH on imaging turned out to be HCA/HCC on pathology, in one of these patients the treatment recommendation formulated by the expert radiologists was to biopsy the lesion. Thus, the sensitivity of imaging for the diagnosis of FNH was 96.9% (95% CI: 90.7–99.5%) and the specificity was 93.8% (95% CI: 86.1–98.0%). The positive predictive value was 93.9% (95% CI: 86.5–98.1%) and the negative predictive value was 96.8% (95% CI: 90.4–99.5%).

### Imaging characteristics exclusively occurring in HCA/HCC

**Fig. 2:**
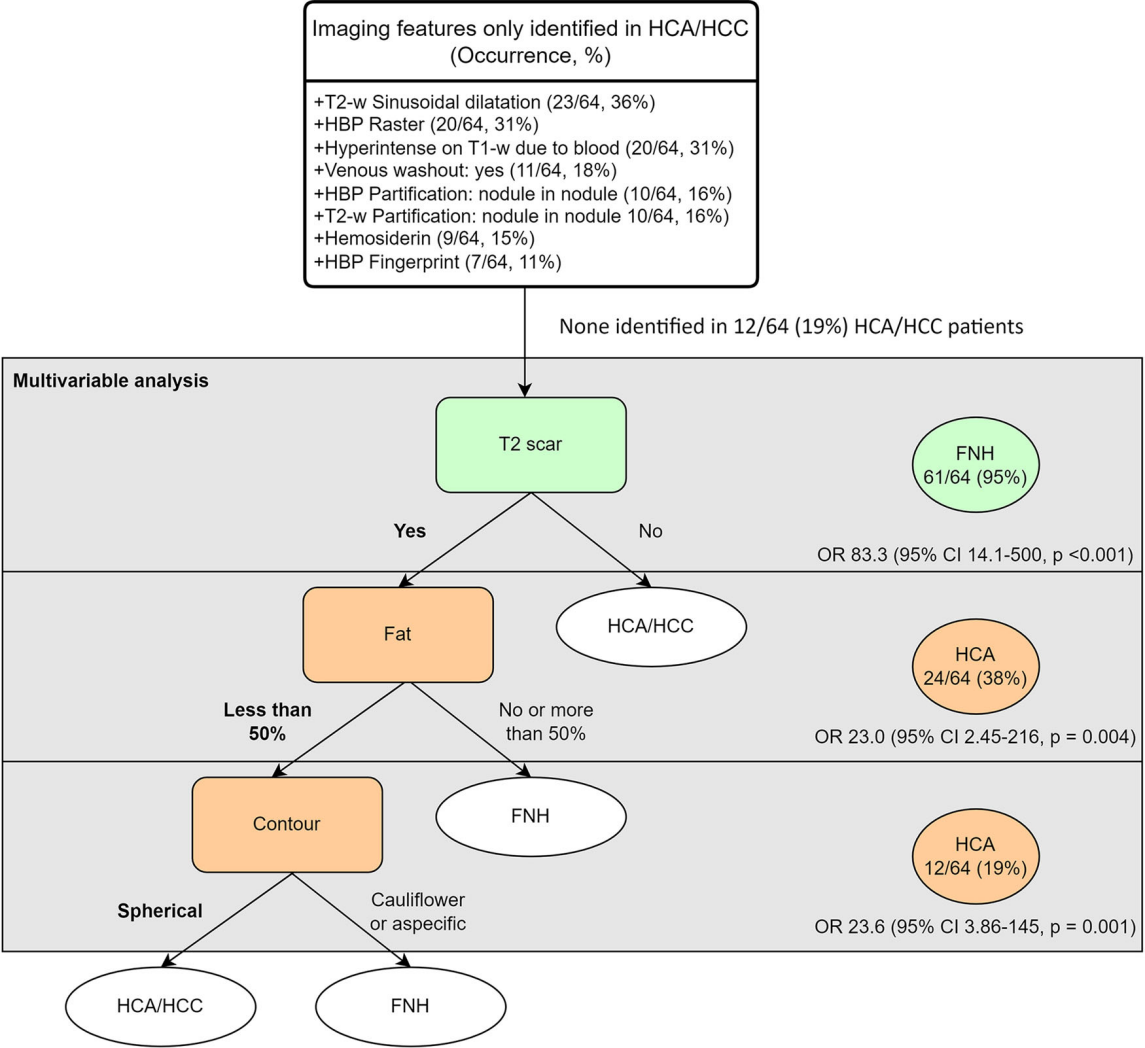
CART and multivariable logistic regression analysis. Figure shows imaging characteristics exclusively identified in HCA/HCC (top part of the figure), and combines the results of the multivariable logistic regression and CART analysis (bottom part of the figure, shaded in gray). The tree structure and division of categorical variables are derived from the CART analysis. The odds ratios are derived from multivariable logistic regression. The occurrence of the identified imaging characteristics in the respective groups is shown in the circles on the right part of the figure

Imaging characteristics were compared between the 64 patients with a pathological diagnosis of FNH and the 64 patients with a pathological diagnosis of HCA/HCC (Table 2). Several imaging characteristics were exclusively observed in HCA/HCC, these were: (i) the presence of a raster pattern in the HBP, (ii) the presence of an atoll fingerprint pattern in the HBP, (iii) the presence of sinusoidal dilatation on T2-w, (iv) the presence of hemosiderin, (v) the presence of T1-w in-phase hyperintensity not due to fat, (vi) the presence of venous washout, and (vii) the presence of a nodule in nodule participation in the HBP and on T2-w (Fig. 2).

**Table 2:** Occurrence and univariable analysis of imaging characteristics in FNH and HCA/HCC

	Pathology diagnosis, (*n* %)		Univariable analysis OR, (95% CI)	*p*-value
	FNH, (*n* = 64)	HCA/HCC, (*n* = 64)		
HBP pattern				
Extensive part hyper-/isointense	59 (92)	37 (58)	*0.116 (0.041–0.328)*	< 0.001
Extensive heterogeneity	9 (14)	25 (39)	**3.92 (1.65–9.31)**	0.001
Raster	0 (0)	20 (31)	118 (7.14–1938)*	< 0.001
Perilesional hypointense rim	11 (17)	12 (19)	1.11 (0.451–2.74)	0.82
Lesional hyper-/isointense rim	6 (9.4)	4 (6.3)	0.644 (0.173–2.40)	0.51
Atoll fingerprint	0 (0.0)	7 (11)	32.5 (1.92–552)*	0.01**
White-bordered flower	29 (45)	2 (3.1)	*0.039 (0.009–0.173)*	< 0.001
Scar	61 (95)	21 (33)	*0.024 (0.007–0.086)*	< 0.001
Liquid	2 (3.1)	18 (28)	**12.1 (2.68–54.9)**	< 0.001
Partification	***0***	***0***		0.003
No	59 (92)	48 (75)	*0.254 (0.087–0.744)*	
­Craquelure	2 (3.1)	0 (0)	0.108 (0.006–2.02)*	
­Focal or mosaic	3 (4.7)	6 (9.4)	2.10 (0.502–8.81)	
Nodule in nodule	0 (0)	10 (16)	48.6 (2.90–812)*	
T2-w pattern				
Extensive heterogeneity	8 (13)	22 (34)	**3.67 (1.49–9.04)**	0.003
Scar	61 (95)	21 (33)	*0.024 (0.007–0.086)*	< 0.001
Liquid	4 (6.3)	22 (34)	**7.86 (2.52–24.5)**	< 0.001
Sinusoidal dilatation	0 (0)	23 (36)	145 (8.81–2383)*	< 0.001
Partification	***0***	***0***		< 0.001
No	63 (98)	49 (77)	*0.052 (0.007–0.406)*	
Focal or mosaic	1 (1.6)	5 (7.8)	5.34 (0.606–47.1)	
Nodule in nodule	0 (0)	10 (16)	48.6 (2.90–812)*	
Miscellaneous				
Arterial hypointense rim	5 (7.8)^1^	14 (22)	**3.25 (1.09–9.65)**	0.03
T1-w in-phase hyperintensity	0 (0)	20 (32)	118 (7.19–1952)*	< 0.001
Hemosiderin	0 (0)	9 (15)^2^	44.6 (2.66–750)*	0.001**
Diffusion restriction	***12***	***11***		0.02
­No	50 (96)	40 (78)	*0.145 (0.030–0.694)*	
Yes	1 (1.9)	3 (5.9)	3.19 (0.320–31.7)	
­Equivocal	1 (1.9)	8 (16)	**9.49 (1.14–78.9)**	
Venous washout	***0***	***1***		0.001
No	60 (94)	45 (71)	*0.167 (0.053–0.527)*	
Yes	0 (0)	11 (18)	55.3 (3.32–923)*	
Pseudo	4 (6.3)	4 (6.3)	1.02 (0.243–4.26)	
Equivocal	0 (0)	3 (4.8)	13.9 (0.773–249)*	
Fat	***1***	***1***		0.009
­No	55 (87)	49 (78)	0.509 (0.197–1.32)	
­Less than 50%	2 (3.2)	12 (19)	**7.18 (1.54–33.6)**	
More than 50%	6 (9.5)	2 (3.2)	0.311 (0.060–1.61)	
Contour	***0***	***0***		< 0.001
Spherical	3 (4.7)	24 (38)	**12.2 (3.44–43.2)**	
­Cauliflower	30 (47)	1 (1.6)	*0.018 (0.002–0.138)*	
Aspecific	31 (48)	39 (61)	1.66 (0.823–3.35)	

Missing values are not included in percentages. Missing values are given in superscript in dichotomous imaging features. Missing values are given in bold italic for categorical imaging features. Odds ratios are calculated for each subcategory of categorical imaging features, and *p*-values of categorical imaging features are given for each imaging feature and not for each subcategory of the imaging feature. Values in bold indicate imaging characteristics occurring more frequently in HCA/HCC (statistically significant). Values in italics indicate imaging characteristics occurring more frequently in FNH (statistically significant)

* Odds ratios obtained by Haldane–Anscombe correction

** Fisher’s exact test

**Fig. 3:**
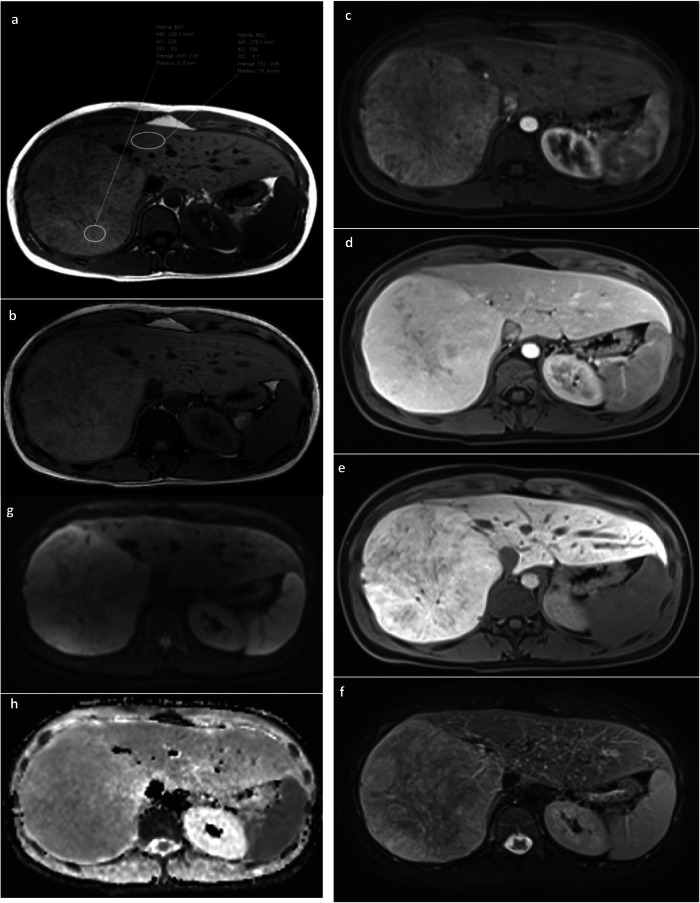
Case of a patient with imaging features suggestive of FNH occurring in a B-catenin-activated HCA. Large liver mass (B-catenin inflammatory HCA, exon 3, non-S45) in a 21-year-old female using oral contraceptives. On T1-w in-phase and out-of-phase imaging (**a**, **b**), we can appreciate a slightly intrinsically hyperintense tumor (226 vs 186 units) which is hypervascular during the arterial and venous phase (**c**, **d**). During the HBP (20 min) (**e**), the tumor is clearly hyper- to isointense to the surrounding liver. Mainly in the center of the lesion, some hypointense lines can be seen, which possibly comprise scar tissue. On T2-w imaging with fat suppression a large rim of hyperintense signal can be visualized and central inhomogeneity (**f**). This was classified as sinusoidal dilatation with possible atoll configuration. There was no restriction on the apparent diffusion coefficient map (**g**, **h**). The combination of extensive hyper- or isointense signal in the HBP (typical for FNH), together with features very typical or unequivocal for HCA/HCC (sinusoidal dilatation and T1-w intrinsic hyperintensity) should prompt a biopsy. The presence of a scar (which is typical for FNH) should not mislead the reader in this situation

Supplementary Table 3 shows imaging characteristics comparing HCA and HCC: (i) up to (iv) were indicative of HCA, and (v) up to (vii) of HCC. Figure 3 shows a case of a patient with imaging characteristics specific to HCA/HCC. In 12/64 (19%) HCA/HCC patients, none of these imaging characteristics were observed. Ten out of twelve were HCA, one was an uncertain HCA/HCC based on immunohistochemistry, and one was an HCC in a male patient based on morphology.

### Imaging characteristics suggestive of FNH or HCA/HCC

Other imaging characteristics were suggestive of, yet not specific for, FNH or HCA/HCC. The sensitivity, specificity, positive predictive value, and negative predictive value of (subcategories of) these imaging characteristics are shown in Table 3. These imaging characteristics were included in multivariable analyses (Supplementary Table 4).

**Table 3:** Imaging features exclusively seen in HCA/HCC and/or suggestive for FNH of HCA/HCC

Imaging features exclusively seen in HCA/HCC	
Dichotomous imaging features	OR (95% CI)
HBP raster	118 (7.14–1938)
HBP atoll fingerprint	32.5 (1.92–552)
T2-w sinusoidal dilatation	145 (8.81–2383)
T1-w in-phase hyperintensity	118 (7.19–1952)
Hemosiderin	44.6 (2.66–750)
**Categorical imaging features**	**OR (95% CI)**
HBP partification: nodule in nodule	48.6 (2.90–812)
T2-w partification: nodule in nodule	48.6 (2.90–812)
Venous washout: yes	55.3 (3.32–923)

Diagnostic performance of imaging features suggestive for FNH					
Dichotomous imaging features	Sens, 95% CI	Spec, 95% CI	PPV, 95% CI	NPV, 95% CI	1/OR (95% CI)*
HBP extensive part hyper-/isointense	92.2 (84.0–97.1)	42.2 (30.6–54.4)	61.5 (51.5–70.8)	84.4 (69.4–94.1)	8.62 (3.05–24.4)
HBP white-bordered flower	45.3 (33.5–57.5)	96.9 (90.7–99.5)	93.5 (81.4–98.9)	63.9 (54.1–73.0)	25.6 (5.78–111)
HBP scar	95.3 (88.3–98.8)	67.2 (55.2–77.9)	74.4 (64.3–83.0)	93.5 (84.0–98.3)	41.7 (11.6–143)
T2-w scar	95.3 (88.3–98.8)	67.2 (55.2–77.9)	74.4 (64.3–83.0)	93.5 (84.0–98.3)	41.7 (11.6–143)

Categorical imaging features	Sens, 95% CI	Spec, 95% CI	PPV, 95% CI	NPV, 95% CI	1/OR (95% CI)
HBP partification: no	92.2 (84.0–97.1)	25.0 (15.5–36.5)	55.1 (45.7–64.4)	76.2 (55.6–90.7)	3.94 (1.34–11.5)
T2-w partification: no	98.4 (93.3–99.9)	23.4 (14.2–34.7)	56.3 (47.0–65.2)	93.8 (75.3–99.6)	19.2 (2.46–143)
Diffusion restriction: no	96.2 (88.6–99.3)	21.6 (11.8–34.1)	55.6 (45.2–65.6)	84.6 (59.6–97.3)	6.90 (1.44–33.3)
Venous washout: no	93.8 (86.1–98.0)	28.6 (18.4–40.4)	57.1 (47.6–66.4)	81.8 (62.7–94.0)	5.99 (1.90–18.9)
Contour: cauliflower	46.9 (35.0–59.0)	98.4 (93.3–99.9)	96.8 (86.6–99.8)	64.9 (55.2–74.0)	55.5 (7.25–500)

Diagnostic performance of imaging features suggestive for HCA/HCC					
Dichotomous imaging features	Sens, 95% CI	Spec, 95% CI	PPV, 95% CI	NPV, 95% CI	OR (95% CI)
HBP extensive heterogeneity	39.1 (27.7–51.3)	85.9 (76.1–93.0)	73.5 (57.3–86.3)	58.5 (48.4–68.1)	3.92 (1.65–9.31)
HBP liquid	28.1 (18.1–39.9)	96.9 (90.7–99.5)	90.0 (72.2–98.3)	57.4 (48.0–66.5)	12.1 (2.68–54.9)
T2-w extensive heterogeneity	34.4 (23.5–46.5)	87.5 (78.0–94.1)	73.3 (56.0–86.8)	57.1 (47.3–66.7)	3.67 (1.49–9.04)
T2-w liquid	34.4 (23.5–46.5)	93.8 (86.1–98.0)	84.6 (67.8–94.9)	58.8 (49.1–68.1)	7.86 (2.52–24.5)
Arterial hypointense rim	21.9 (13.0–33.0)	92.1 (83.7–97.1)	73.7 (51.7–89.7)	53.7 (44.3–62.9)	3.25 (1.09–9.65)

Categorical imaging features	Sens, 95% CI	Spec, 95% CI	PPV, 95% CI	NPV, 95% CI	OR (95% CI)
Diffusion restriction: equivocal	15.7 (7.5–27.2)	98.1 (91.8–99.9)	88.9 (59.5–99.3)	54.3 (44.2–64.1)	9.49 (1.14–78.9)
Fat: less than 50%	19.0 (10.7–29.9)	96.8 (90.5–99.5)	85.7 (62.1–97.5)	54.5 (45.2–63.5)	7.18 (1.54–33.6)
Contour: spherical	37.5 (26.3–49.7)	95.3 (88.3–98.8)	88.9 (73.7–97.1)	60.4 (50.7–69.6)	12.2 (3.44–43.2)

Odds ratios obtained by Haldane–Anscombe correction, no sensitivity, specificity, PPV, or NPV calculated due to zero-cell counts

* Inverse of the odds ratio is given, this improves the comparison of odds ratios with odds ratios of imaging features suggestive of HCA/HCC

Excluded from multivariable analyses were HBP partification, HBP scar, and HBP liquid, because these imaging features had a correlation efficiency of 0.80 or higher with their T2-w counterparts. Perilesional hypointense rim and lesional hyper-/isointense rim were excluded because these were not associated in the univariable analysis, and diffusion restriction was excluded due to missing data.

The outcomes of regression tree analysis and multivariable logistic regression were similar (CART *R*^2^ = 0.766 and Nagelkerke *R*^2^ = 0.844, respectively). Three additional imaging features that were independently associated with either FNH or HCA/HCC were identified (Fig. 2): (i) the presence of a scar on the T2-w, indicating FNH, (ii) the presence of less than 50% fat, indicating HCA/HCC, and (iii) a spherical contour indicating HCA/HCC.

### Occurrence of imaging features suggestive of FNH in B-catenin-activated HCA

The presence of a T2-w scar, which was associated with FNH in the multivariable analysis and was present in 61/64 (95%) of FNH, was also present in 4/14 (29%) of B-catenin-activated HCA and in two out of five B-catenin activated HCA with a non-S45 exon 3 mutation (Table 4 and Supplementary Table 5).

**Table 4:** Occurrence of the imaging features suggestive of FNH in B-(I)HCA

	Occurrence in FNH, (*n* = 64)	Occurrence in B-(I)HCA, (*n* = 14)	Occurrence in B-(I)HCA with non-S45 exon 3 mutations, (*n* = 5)
Dichotomous imaging features			
HBP extensive part hyper-/isointense	59 (92)	13 (93)	5 (100)
HBP white-bordered flower	29 (45)	1 (7.1)	0 (0)
HBP scar	61 (95)	5 (36)	2 (40)
T2-w scar	61 (95)	4 (29)	2 (40)
Categorical imaging features			
HBP partification: no	59 (92)	13 (93)	5 (100)
T2-w partification: no	63 (98)	14 (100)	5 (100)
Diffusion restriction: no	50 (96)	12 (100)	4 (100)^1^
Venous washout: no	60 (94)	13 (93)	5 (100)
Contour: cauliflower	30 (47)	1 (7.1)	0 (0)

B-(I)HCA is a subgroup of HCA. B-(I)HCA with non-S45 exon 3 mutations is a subgroup of B-(I)HCA

Missing values are given in superscript

Other imaging features suggestive of FNH that occurred in almost all FNH were also frequently present in B-catenin-activated HCA, i.e., extensive hyper-/isointensity, the absence of partification, the absence of diffusion restriction, and the absence of venous washout. Cauliflower contour and HBP White-bordered flower were both rarely observed (1/14, 7.1%) in B-catenin activated HCA, yet were also present in less than 50% of FNH. Figure 3 shows a case of a patient with imaging features suggestive of FNH occurring in a B-catenin-activated HCA.

## Discussion

This multicenter study found that imaging has high sensitivity and specificity for differentiation of FNH and HCA/HCC hyper- or isointensity in the HBP. In more than 80% of HCA/HCC, at least one of the imaging features that occurred exclusively in HCA/HCC was present. These features, with acceptable intra- and interobserver reliability, aid the clinically relevant distinction between FNH and HCA/HCC. The absence of these highly specific features mostly occurred in HCA.

Comparing the imaging characteristics suggestive of HCA/HCC identified in this study to the literature, the presence of sinusoidal dilatation on T2-w has been described in inflammatory HCA and rarely in HCC [23, 34]. Some other imaging features were also described for the Liver Imaging Reporting and Data System criteria for HCC, for example, the presence of nodule-in-nodule partification in the HBP and on T2-w [9, 25, 35]. Venous washout exclusively occurred in HCA/HCC, although assessment of venous washout is hampered in lesions hyper- to isointense in the HBP by early uptake of Gd-EOB-DTPA by hepatocytes [36]. In addition, the study identified novel features indicating HCA/HCC, including the raster and atoll fingerprint pattern in the HBP; and features indicating FNH, including the white-bordered flower.

Regarding the imaging diagnosis of FNH, according to EASL guidelines, the diagnosis is generally made by a set of characteristics indicative of FNH, and the absence of characteristics suggesting HCA/HCC warranting further analysis [4]. In multivariable analyses, a T2-w scar was independently associated with FNH with an odds ratio of more than 80. Yet, the confidence interval of the odds ratio was wide. In the absence of other imaging features completely specific to FNH, caution is advised. Undertreatment of HCA/HCC caused by misdiagnosis as FNH could result in malignant transformation [4, 37, 38].

Our study, which is one of the first to assess B-catenin mutated HCA alongside a control group of FNH, contributes to the expanding body of evidence that suggests that the threshold for biopsy and/or resection should perhaps be lowered for lesions showing HBP hyper- or isointensity. Lesions with imaging characteristics exclusively seen in or suggestive of HCA/HCC should be suspected to be HCA/HCC despite the presence of extensive hyper- or isointensity in the HBP and/or a T2-w scar. A T2-w scar was independently associated with FNH in the multivariable analysis, but was also present in 4/14 (29%) of B-catenin-activated HCA. These findings are in line with previous studies that have shown that B-catenin-mutated HCA may show a T2-w scar [39]. Other imaging characteristics suggestive of FNH such as the absence of venous washout or diffusion restriction were also frequently present in B-catenin-activated HCA. Other authors have suggested quantitative HBP analysis to differentiate FNH from HCA/HCC [37]. Our study confirms that hyper- or isointensity in the HBP has a high specificity for FNH, as all included FNH were hyperintense. However, in the current cohort and in previous literature, B-catenin-activated HCA is frequently extensively hyper- or isointense (13/14 B-catenin activated HCA) [40]. In our study, almost half of the HCA and HCC (95/211, 45% of HCA and 43/90, 48% of HCC) were also hyper- or isointense in the HBP, which is much higher than reported in previous studies [1, 10, 14, 15]. Selection bias in patients with HCC who underwent Gd-EOB-DPTA enhanced imaging and in patients with HCA who underwent biopsy and/or resection may be present. Nonetheless, it is striking that 14/48 (29%) of HCA were B-catenin mutated HCA.

This study has some limitations. First, diffusion-weighted imaging was excluded from multivariable analysis because these were often missing due to insufficient quality. Second, the method to characterize B-catenin-activated HCA provided high specificity for B-catenin-activated HCA, but may lack sensitivity [41, 42]. This may have introduced bias as exclusively lesions with strong B-catenin activation (indicated by overexpression of GS-staining) may have been included and limits the number of B-catenin activated HCA identified in the current study [42]. Third, the retrospective study design is prone to selection bias and the sample size may lead to overfitting of the multivariable logistic regression analysis. Thus, caution is advised when extrapolating the imaging characteristics that were exclusively observed in FNH or HCA/HCC in our study to larger and other populations. In addition, external validation of the CART analysis is warranted. Fourth and finally, the differentiation with other types of potentially hyper- or isointense liver lesions was not assessed. These other lesions were excluded from this study as these are hyper- or isointense not due to intracellular uptake in hepatocytes, but through other mechanisms [1].

In conclusion, our study provides a diagnostic algorithm with potential clinical application. For lesion hyper- or isointense in the HBP, raster or atoll fingerprint, sinusoidal dilatation, hemosiderin, T1-w hyperintensity, and/or nodule-in nodule partification should lead to suspicion of HCA/HCC. If these lesions are extensively hyper- to isointense in the HBP, further investigation may be warranted.

## Supplementary information

ELECTRONIC SUPPLEMENTARY MATERIAL

## Abbreviations

CART: Classification and regression tree
FNH: Focal nodular hyperplasia
Gd-EOB-DTPA: Gadolinium-ethoxybenzyl-diethylenetriamine pentaacetic acid
HBP: Hepatobiliary phase
HCA: Hepatocellular adenoma
HCC: Hepatocellular carcinoma
MRI: Magnetic resonance imaging
