# Supplementary material for: Lesions hyper- to isointense to surrounding liver in the hepatobiliary phase of gadoxetic acid-enhanced MRI
Source: Eur Radiol. 2024 Jun 20;34(12):7661–72. doi: 10.1007/s00330-024-10829-x (PMC11557620; doi:10.1007/s00330-024-10829-x)
Supplement: Supplementary file 1 — ELECTRONIC SUPPLEMENTARY MATERIAL [file 330_2024_10829_MOESM1_ESM.pdf]

Lesions hyper- to isointense to the surrounding liver in the hepatobiliary phase of gadoxetic acid enhanced MRI

ELECTRONIC SUPPLEMENTARY MATERIAL

Supplementary File 1. Signs and explanations

Hepatobiliary phase (HBP):

1. **HBP-Hyper- or isointensity**: The extent of HBP hyper- or isointensity was defined as limited or extensive, based on visual assessment.
2. **HBP-Heterogeneity:** The extent of HBP heterogeneity was defined as no/limited or extensive, based on visual assessment of uptake and excluding heterogeneity caused by other imaging patterns. Figure (right) shows extensive HBP heterogeneity.

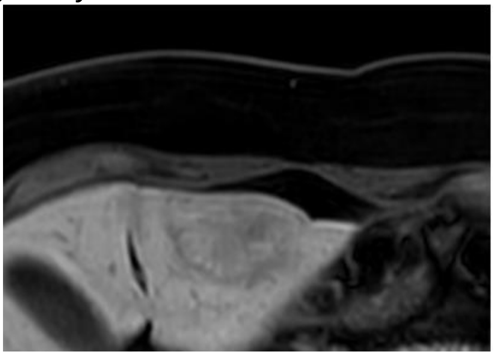

3. **HBP-Raster**: Demarcated regions in the tumor with typical appearance small lines with increased uptake of Gd-EOB-DTPA in the HBP. The background has typically less uptake of Gd-EOB-DTPA. The lines may intersect with each other. Sometimes these lines have a more peripheral

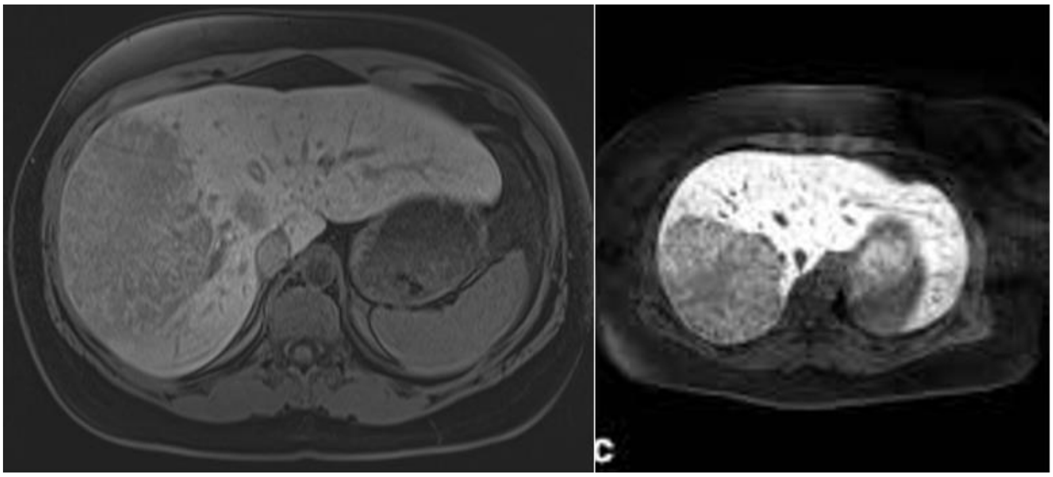

predominance. Two figures (below) show peripherally oriented raster on HBP.

4. **HBP-Perilesional hypointense rim**: Sharply demarcated region around the tumor with typically less uptake of Gd-EOB-DTPA. This feature is suggestive of a capsule surrounding the tumor. This small rim is different from the targetoid appearance which is a feature from inside the tumor and often larger in size.[1] Figure (right) shows dark rim surrounding the tumor.

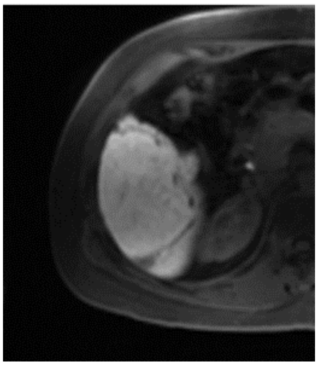

5. **HBP-Lesional hyper- or isointense rim**: Sharply demarcated region at the periphery of the tumor with increased uptake of Gd-EOB-DTPA in the HBP. This region has typically a center part with less uptake of Gd-EOB-DTPA. It is thought to be a typical pattern for focal nodular hyperplasia. In these situations the rim represents intracellular uptake of Gd-EOB-DTPA and the center part represents scar tissue. The scar tissue is typically not linear but diffuse.[2] Figure (right) shows example of hyperintense rim on HBP.

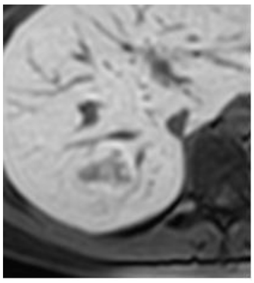

6. **HBP-Atoll fingerprint**: Demarcated regions in the tumor with different uptake comparable to a fingerprint of the atoll-appearance of the tumor on T2-weighted imaging. This sign is therefore only present in tumors with a typical atoll-sign on T2-weighted imaging. T2-w image with atoll

configuration (left, below) and atoll fingerprint on HBP (right, below).

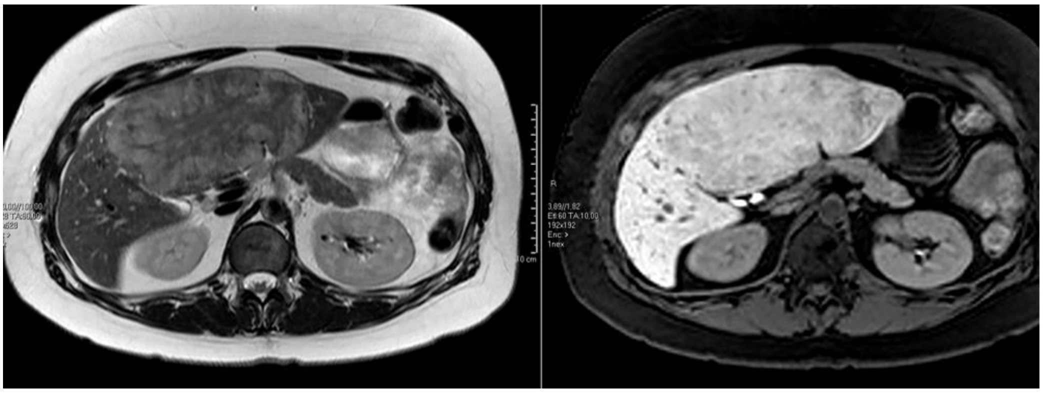

7. **HBP-White-bordered flower**: Demarcated regions in the tumor with different uptake of Gd-EOB-DTPA. This pattern is recognizable by the presence of diffuse uptake of Gd-EOB-DTPA except by a central part of less uptake with a scar appearance and a region of borderline less uptake (probably also scar tissue) in the region between the central part and the periphery. The peripheral region has a more rim-like appearance of increased uptake. Furthermore, the tumor typically has a lobulated appearance. The overall appearance is most comparable with a white bordered flower as shown in figure. Two examples of white bordered flower in HBP, and source of the name of this imaging pattern (right).

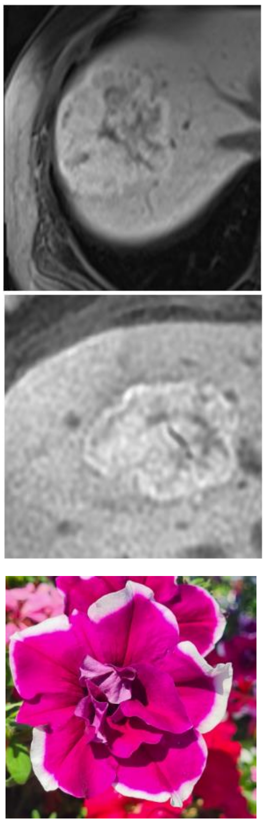

8. **HBP-Scar**: Demarcated region in the tumor with less uptake of Gd-EOB-DTPA in the hepatobiliary phase. This region can be present anywhere in the tumor, but often typically in the central part. It often has a linear appearance or spoke wheel aspect, however, scar tissue can also be a more diffuse region with a typical appearance: combination with hyperintensity on T2-weighted imaging, hypointensity on T1-weighted imaging and hypointensity in the transitional phase (3-5 minutes after contrast injection).[3] Two lesions in the liver with extensive uptake except for the scartissue which remains hypointense (right).

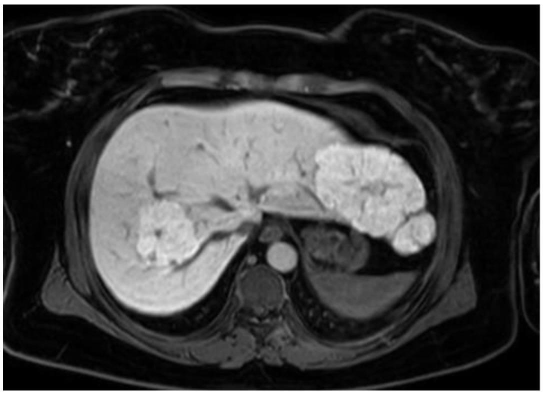

9. **HBP-Liquid**: Sharply demarcated region with no uptake of Gd-EOB-DTPA. By comparing this region with T2-weighted imaging this region is stated as liquid (i.e. high intensity on T2-weighted imaging). This liquid region can originate from water or blood. In latter situation, this part is often intrinsically hyperintense on T1-weighted imaging due to blood degradation products. Example of a lesion with localized absence of uptake (right). This is the same lesion the image for T2-w liquid.

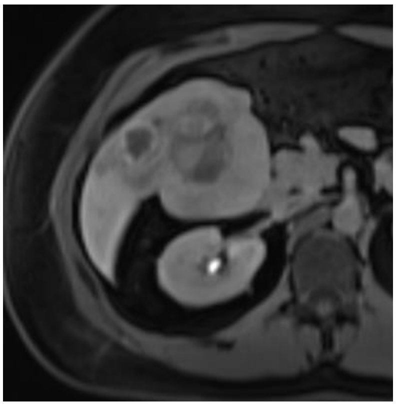

10. **HBP-Partification**: Sharply demarcated regions in the tumor with different uptake of Gd-EOB-DTPA in the hepatobiliary phase. Three different

subtypes are described: craquelure with loosening part (abbreviated to craquele), focal or mosaic architecture, and nodule-in-nodule.

a. **Craquelure with loosening part**: Subtype of partification in the HBP. This subtype is recognizable by the combination hypointense lines (probably scar tissue) intersecting each other on a background of hyper- or isointense uptake with typical loosening parts. These loosening parts comprise of regions between the hypointense lines which are not hyper- or isointense. This is comparable to an old painting with craquelure with loosening parts as appreciable on the image. Two examples of craquelure with loosening parts in HBP (below).

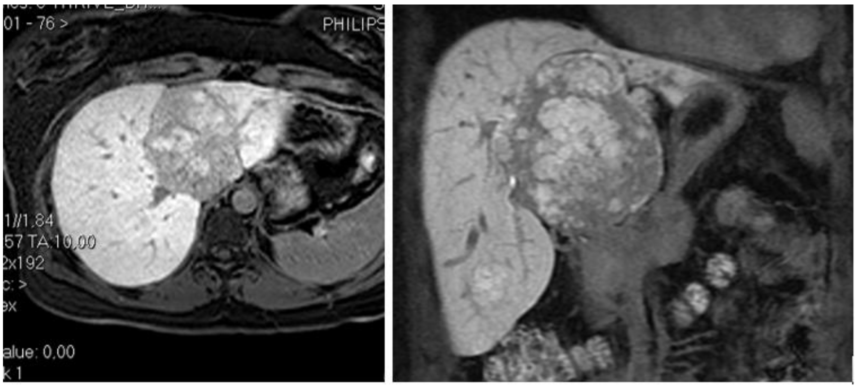

b. **Focal or mosaic architecture**: Subtype of partification in the HBP. This subtype is characterized by a demarcated focal non-nodular different uptake in the tumor. This is due to multiple histological components resembling multicolor tiles of a mosaic.[4] One example

of mosaic architecture representing different non-nodular uptake (below)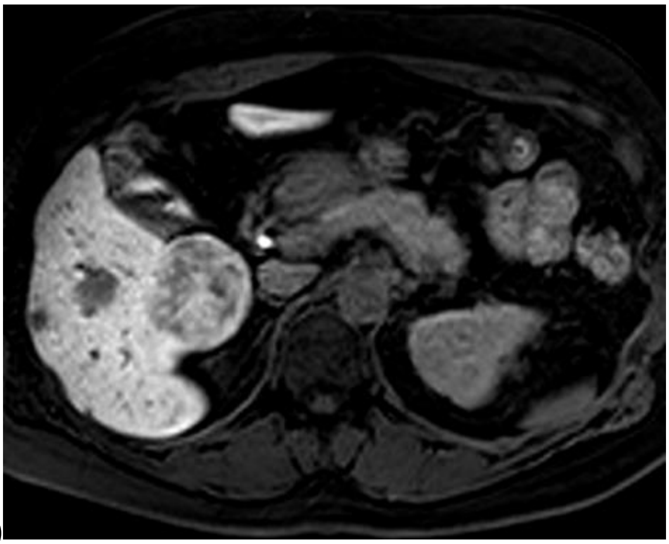

c. **Nodule-in-nodule**: Subtype of partification in the HBP. This subtype is comparable to the focal/mosaic pattern, with the exception that the focal demarcation is nodular.[5] Typical example of nodule-in-nodule (below).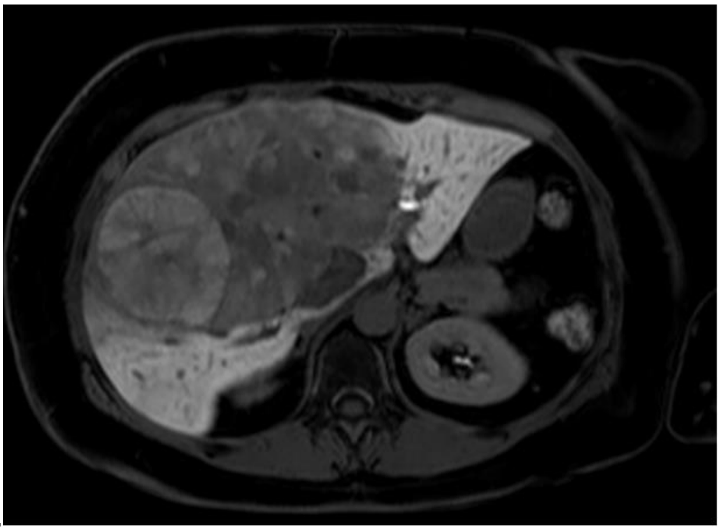

T2-weighted imaging patterns

1. **T2w-heterogeneity:** The extent of T2w-heterogeneity was defined as no/limited or extensive, based on visual assessment and excluding heterogeneity caused by other imaging patterns. First example of limited

heterogeneity (below, left) and second example of extensive heterogeneity (below, right).

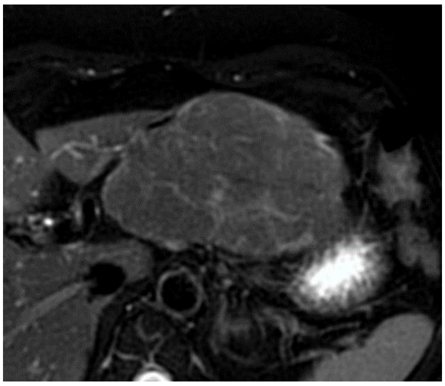 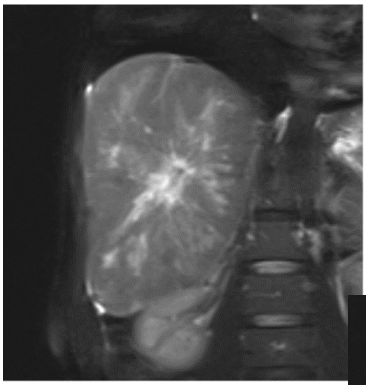

2. **T2w-Scar**: Demarcated region in the tumor with hyperintense signal on T2-weighted imaging. This region can be present anywhere in the tumor, but often typically in the central part. It often has a linear appearance or spoke wheel aspect, however, scar tissue can also be a more diffuse region with a typical appearance: combination with hyperintensity on T2-weighted imaging, hypointensity on T1-weighted imaging and hypointensity in the transitional phase (3-5 minutes after contrast injection).[3] Typical example of a T2-w hyperintense scar (right).

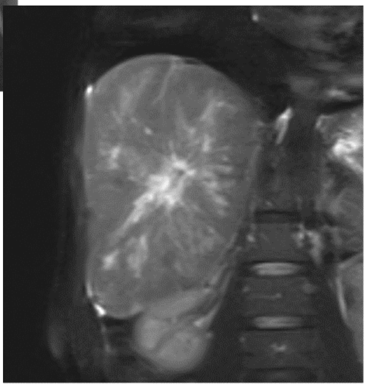

3. **T2w-Liquid**: Sharply demarcated strongly hyperintense region on T2-weighted imaging. This liquid region can originate from water or blood. In latter situation this part is often intrinsically hyperintense on T1-weighted imaging due to subacute blood degradation products. Example of liquid parts in liver lesion on T2-w imaging (right).

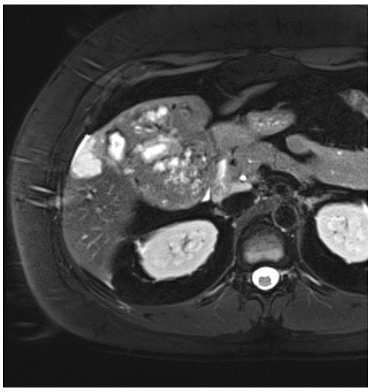

4. **T2w-Sinusoidal dilatation**: Demarcated hyperintense regions in the tumor. The pattern can be like an atoll, or more diffuse throughout the tumor . The atoll sign was described by van Aalten *et al*. as a hyperintense

rim in the periphery of the lesion on T2-weighted imaging with an isointensity in the center of the lesion reminiscent of the sea within an atoll.[6] Small intralesional T2-hyperintense nodules can be found in the center of the lesion (small islands). Two particular subtypes are the crescent sign and a new sign ‘the black atoll’. The first is described by Bise *et al.* as: a peripheral portion of hyperintense rim on T2-weighted and/or arterial phase with persistent delayed enhancement especially in tumors with steatotic or remodeled center.[7] In latter subtype, the central parts of the tumor which normally are isointense to the surrounding liver are now strongly hypointense probably due to diffuse hemosiderin deposition. Typical example of an atoll sign on T2-w imaging including central islands (below, left). Second image shows a typical case of a black atoll. The part which is normally isointense to the surrounding liver is dark due to hemosiderin (below, right).

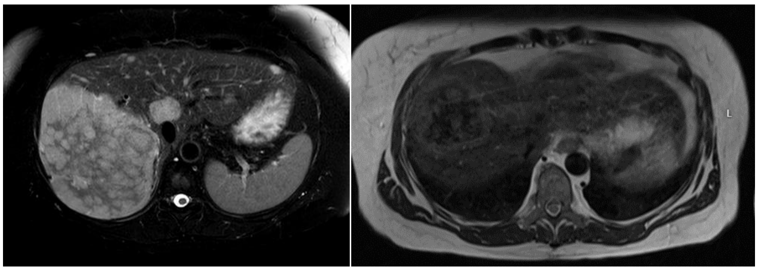

5. **T2w-Partification:** Sharply demarcated regions in the tumor with different intensity on T2-weigthed imaging. Two different subtypes are described: focal or mosaic architecture, and nodule-in-nodule.

a. **Focal or mosaic architecture**: Subtype of partification on T2-weighted imaging. This subtype is characterized by a demarcated focal non-nodular intensity in the tumor. This is due to multiple histological components resembling multicolor tiles of a mosaic.[4] Typical example of focal mosaic pattern on T2-w imaging (right).

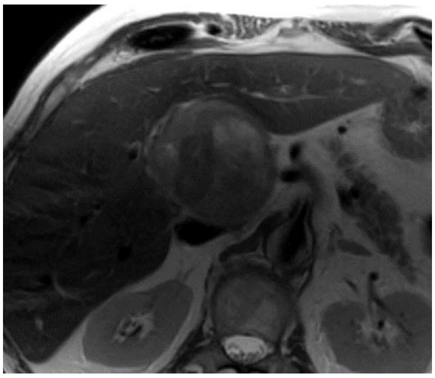

b. **Nodule-in-nodule**: Subtype of partification on T2-weighted imaging. This subtype is comparable to the focal/mosaic pattern, with the exception that the focal demarcation is nodular.[5] Typical example of nodule in nodule on T2-w imaging (right).

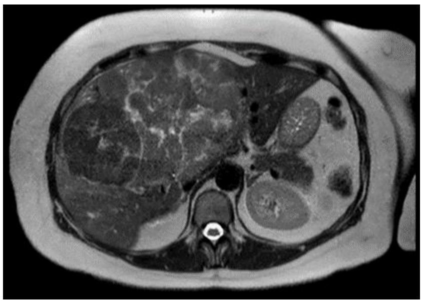

Miscellaneous:

1. **Arterial hypointense rim**: Presence of a continuous demarcated rim of non-enhancement at the periphery of an arterial hypervascular tumor. Example of hypointense rim in arterial phase (right).

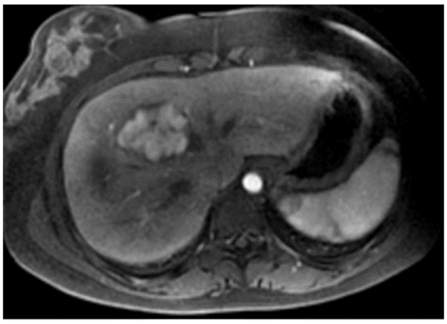

2. **T1-w in-phase hyperintensity not due to fat**: Containing regions hyperintense on T1-weighted in-phase imaging due to subacute hemorrhage. Fat may also be hyperintense on in-phase T1-w, which is excluded by comparing the in-phase and out-of-phase sequences (in case of fat, there is signal loss in out-of-phase compared to in-phase). Example of intrinsic hyperintensity

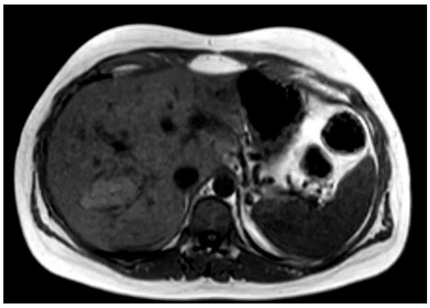

in in-phase T1-w imaging (right). Abbreviated to T1-w in-phase hyperintensity

3. **Hemosiderin**: Containing regions hypointense on, T1-w and T2-w weighted imaging, and blooming on out-of-phase T1-weighted imaging and diffusion weighted imaging. Same lesion with the black atoll sign. Remark the blooming on the in-phase imaging due to longer echo time (below).

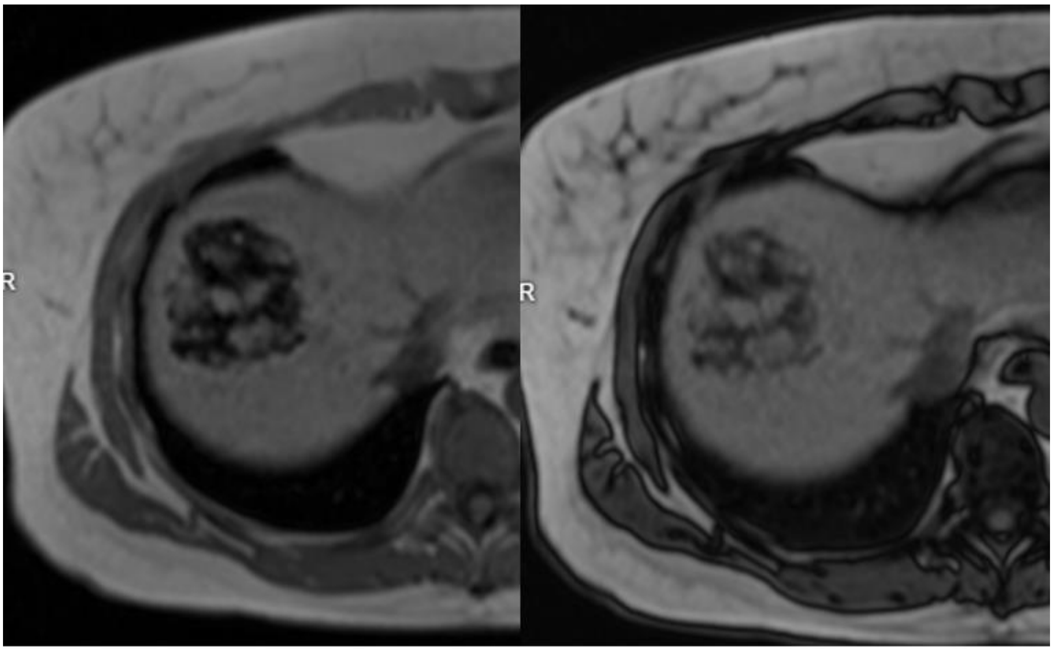

4. **Diffusion restriction:** evaluable by apparent diffusion coefficient with high b value equal to or above 600 s/mm^2. Diffusion restriction is present if the apparent diffusion coefficient (ADC) is lower in the tumor than in the non-lesional surrounding liver parenchyma.
5. **Venous washout:** To determine whether wash-out was still evaluable, the intensity of the portal vein was compared to adjacent liver. Washout was deemed evaluable if the portal vein was hyper- or isointense relative to the adjacent liver.[8-10] Although this technique is not scientifically validated, no other cut-off is currently validated as reliable.

6. **Fat:** defined as absent, less than fifty per cent of the lesion, and more than fifty per cent of the lesion. Containing area, typically hyper-/isointense on T1-w imaging, with more signal loss in out-of-phase compared to in-phase.
7. **Contour**
   a. **Spherical:** Tumor with a round or oval contour, without lobulated contour. Typical example of an oval liver tumor on T2-w imaging (right).

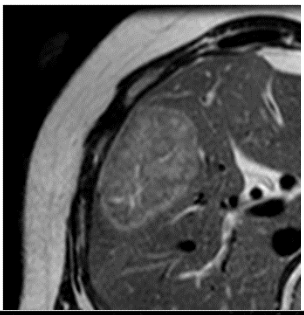

   b. **Cauliflower**: Tumor with a multilobulated appearance like a cauliflower, in which each lobule has its own core. The difference with nodule-in-nodule is that nodule-in-nodules have different signal properties. Example of a cauliflower presentation on T1-w imaging in HBP (right).

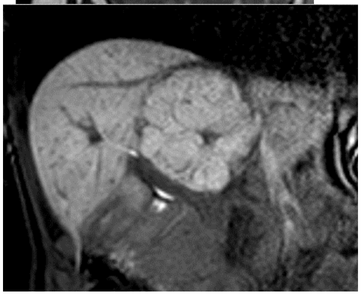

   c. **Aspecific:** Tumor with a lobulated contour which has one central core.

Supplementary File 2. STROBE Statement—checklist of items that should be included in reports of observational studies

	Item No.	Recommendation	Page No.	Relevant text from manuscript
**Title and abstract**	1	(*a*) Indicate the study's design with a commonly used term in the title or the abstract	2	"A multicenter retrospective cohort study…"
		(*b*) Provide in the abstract an informative and balanced summary of what was done and what was found	2	N/A
**Introduction**				
Background/rationale	2	Explain the scientific background and rationale for the investigation being reported	3, 4	N/A
Objectives	3	State specific objectives, including any prespecified hypotheses	3,4	"Therefore, the … enhanced MRI."
**Methods**				
Study design	4	Present key elements of study design early in the paper	5	"A retrospective cohort study…"
Setting	5	Describe the setting, locations, and relevant dates, including periods of recruitment, exposure, follow-up, and data collection	5	"A retrospective … the study."
Participants	6	(*a*) *Cohort study*—Give the eligibility criteria, and the sources and methods of selection of participants. Describe methods of follow-up<br><br>*Case-control study*—Give the eligibility criteria, and the sources and methods of case ascertainment and control selection. Give the rationale for the choice of cases and controls<br><br>*Cross-sectional study*—Give the eligibility criteria, and the sources and methods of selection of participants	5	"A retrospective … were produced."

		(*b*) *Cohort study*—For matched studies, give matching criteria and number of exposed and unexposed<br><br>*Case-control study*—For matched studies, give matching criteria and the number of controls per case	N/A	
Variables	7	Clearly define all outcomes, exposures, predictors, potential confounders, and effect modifiers. Give diagnostic criteria, if applicable	5, 6, Suppl File 1	"Imaging features … storage disorders."
Data sources/ measurement	8*	For each variable of interest, give sources of data and details of methods of assessment (measurement). Describe comparability of assessment methods if there is more than one group	5, 6, Suppl File 1	"Imaging features … storage disorders."
Bias	9	Describe any efforts to address potential sources of bias	5	"All hyper- … pathological classification."
Study size	10	Explain how the study size was arrived at	5	"A retrospective … were produced."
Quantitative variables	11	Explain how quantitative variables were handled in the analyses. If applicable, describe which groupings were chosen and why	6, 7	"Statistical analyses … activated HCA."
Statistical methods	12	(*a*) Describe all statistical methods, including those used to control for confounding	6, 7	"Statistical analyses … activated HCA."
		(*b*) Describe any methods used to examine subgroups and interactions	7	"In univariable … activated HCA."
		(*c*) Explain how missing data were addressed	6, 7	"Statistical analyses … was conducted"
		(*d*) *Cohort study*—If applicable, explain how loss to follow-up was addressed	N/A	

		Case-control study—If applicable, explain how matching of cases and controls was addressed<br><br>*Cross-sectional study*—If applicable, describe analytical methods taking account of sampling strategy		
		(*e*) Describe any sensitivity analyses	7	“Sensitivity analyses … activated HCA.”
**Results**				
Participants	13*	(a) Report numbers of individuals at each stage of study—eg numbers potentially eligible, examined for eligibility, confirmed eligible, included in the study, completing follow-up, and analysed	8, Figure 1	“In the … (Figure 1).”
		(b) Give reasons for non-participation at each stage	8, Figure 1	“In the … (Figure 1).”
		(c) Consider use of a flow diagram	Figure 1	N/A
Descriptive data	14*	(a) Give characteristics of study participants (eg demographic, clinical, social) and information on exposures and potential confounders	8	“The majority … (Table 1).”
		(b) Indicate number of participants with missing data for each variable of interest	Table 2	N/A
		(c) *Cohort study*—Summarise follow-up time (eg, average and total amount)	N/A	
Outcome data	15*	*Cohort study*—Report numbers of outcome events or summary measures over time	N/A	
		Case-control study—Report numbers in each exposure category, or summary measures of exposure	N/A	
		Cross-sectional study—Report numbers of outcome events or summary measures	N/A	

Main results	16	(*a*) Give unadjusted estimates and, if applicable, confounder-adjusted estimates and their precision (eg, 95% confidence interval). Make clear which confounders were adjusted for and why they were included	8, 9, 10, Table 2, Table 3	“In total, …. Table 3.”
		(*b*) Report category boundaries when continuous variables were categorized	N/A	
		(*c*) If relevant, consider translating estimates of relative risk into absolute risk for a meaningful time period	N/A	
Other analyses	17	Report other analyses done—eg analyses of subgroups and interactions, and sensitivity analyses	10, Table 4	“The presence … activated HCA”
**Discussion**				
Key results	18	Summarise key results with reference to study objectives	11	“This study… be determined.”
Limitations	19	Discuss limitations of the study, taking into account sources of potential bias or imprecision. Discuss both direction and magnitude of any potential bias	12	“This study… other mechanisms.”
Interpretation	20	Give a cautious overall interpretation of results considering objectives, limitations, multiplicity of analyses, results from similar studies, and other relevant evidence	11-12	“According to … mutated HCA.”
Generalisability	21	Discuss the generalisability (external validity) of the study results	12	“In our … mutated HCA.”
**Other information**				
Funding	22	Give the source of funding and the role of the funders for the present study and, if applicable, for the original study on which the present article is based	Title page	“This work … for publication.”

*Give information separately for cases and controls in case-control studies and, if applicable, for exposed and unexposed groups in cohort and cross-sectional studies.

Note: An Explanation and Elaboration article discusses each checklist item and gives methodological background and published examples of transparent reporting. The STROBE checklist is best used in conjunction with this article (freely available on the Web sites of PLoS Medicine at http://www.plosmedicine.org/, Annals of Internal Medicine at http://www.annals.org/, and Epidemiology at http://www.epidem.com/). Information on the STROBE Initiative is available at www.strobe-statement.org.

Supplementary Figure 1. Cases of a patient with imaging characteristics specific for HCA/HCC

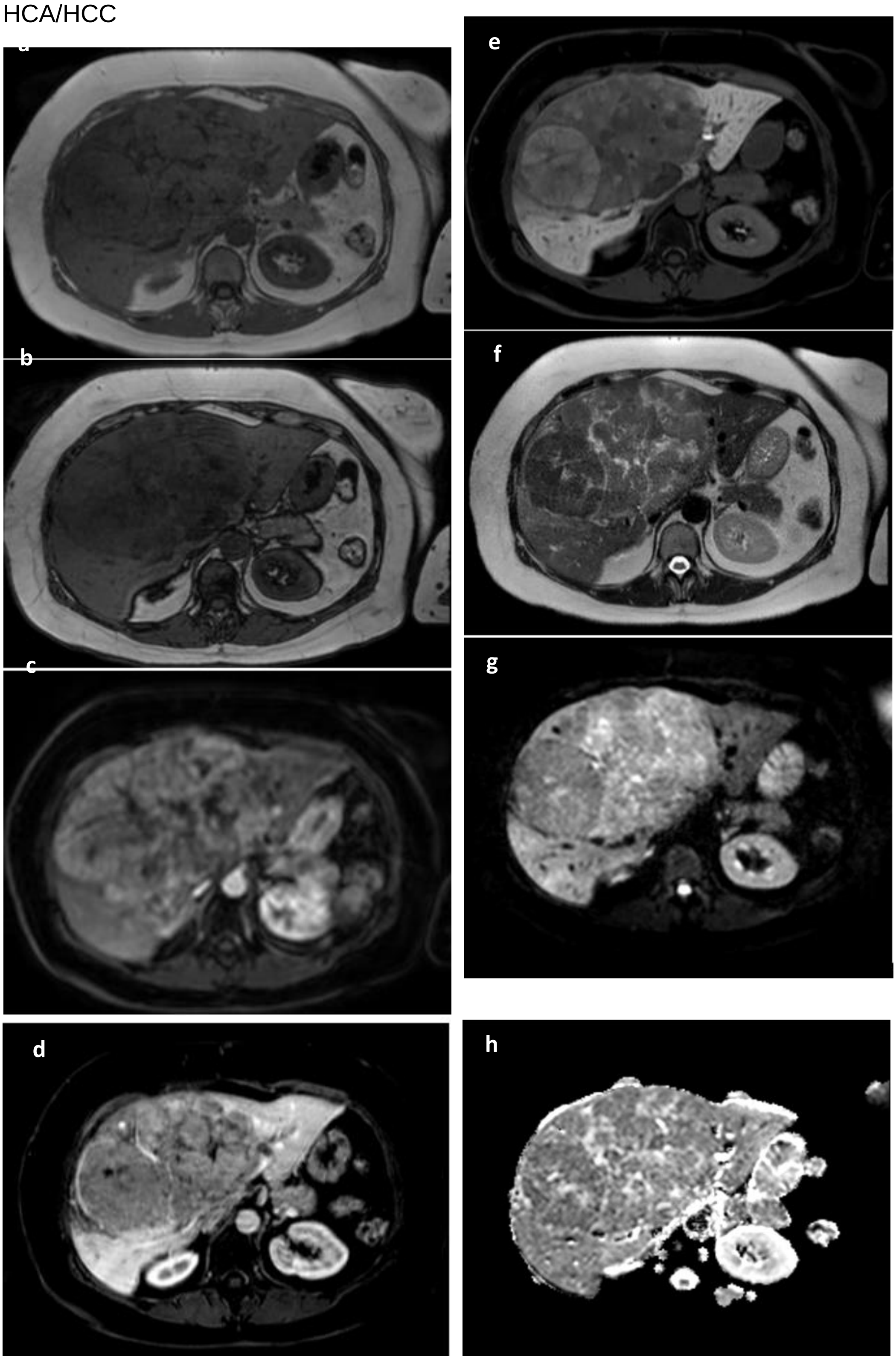

Large liver mass in 35 years old female diagnosed with HCC. Multinodular appearance on T1-w in-phase and out-of-phase with signs of intralesional fat on the out-of-phase (figure 2a-b). Arterial hypervascular tumor (figure 2c) with early wash-out (figure 2d). During the hepatobiliary phase (figure 2e) and on T2-w images (figure 2f sign typicval for nodule-in-nodule can be appreciated. This is differernt from caulifower appearance (typcial for FNH) where multilobular appearance is caused by parts with same appearance on different sequences. Nodule-in-nodule appearance should prompt for biopsy since it is typical for HCC. The appearance on diffucion weighted imaging and ADC (figure 2g-h) are non-specific.

Supplementary Table 1. Interobserver reliability

	Overall		First part		Second part	
**HBP pattern**	n	**Kappa (95%CI)**	n	**Kappa (95%CI)**	**n**	**Kappa (95%CI)**
Extensive part hyper-/isointense	128	0.560 (0.397-0.723)	45	0.450 (0.156-0.744)	83	0.615 (0.427-0.803)
Extensive heterogeneity	128	0.477 (0.312-0.642)	45	0.476 (0.231-0.721)	83	0.381 (0.134-0.628)
Raster	128	0.660 (0.484-0.836)	45	1.000 (1.000-1.000)	83	0.422 (0.157-0.687)
Perilesional hypointense rim	128	0.341 (0.121-0.561)	45	0.404 (0.086-0.722)	83	0.279 (0.005-0.553)
Lesional hyper-/isointense rim	128	0.534 (0.265-0.803)	45	1.000 (1.000-1.000)	83	0.467 (0.173-0.761)
Atoll Fingerprint	128	0.431 (0.029-0.833)	45	0.656 (0.031-1.28)	83	0.320 (-0.156-0.796)
White-bordered flower	128	0.617 (0.458-0.776)	45	0.603 (0.293-0.913)	83	0.614 (0.424-0.804)
Scar	128	0.497 (0.344-0.650)	45	0.571 (0.359-0.783)	83	0.423 (0.219-0.627)
Liquid	128	0.399 (0.162-0.636)	NA	NA	83	0.513 (0.250-0.776)
Partification	128	0.348 (0.176-0.520)	45	0.379 (0.114-0.644)	83	0.349 (0.145-0.553)
**T2-w pattern**	n	**Kappa (95%CI)**	n	**Kappa (95%CI)**	**n**	**Kappa (95%CI)**
Extensive heterogeneity	128	0.404 (0.230-0.578)	45	0.386 (0.116-0.656)	83	0.388 (0.153-0.623)
Scar	128	0.462 (0.311-0.613)	45	0.458 (0.248-0.668)	83	0.439 (0.235-0.643)
Liquid	128	0.378 (0.170-0.586)	45	0.118 (-0.174-0.410)	83	0.484 (0.243-0.725)
Sinusoidal dilatation	128	0.634 (0.467-0.801)	45	0.759 (0.536-0.982)	83	0.546 (0.315-0.777)
Partification	128	0.517 (0.327-0.707)	45	0.464 (0.121-0.807)	83	0.544 (0.317-0.771)
**Miscellaneous**	n	**Kappa (95%CI)**	n	**Kappa (95%CI)**	**n**	**Kappa (95%CI)**
Arterial hypointense rim	128	0.109 (-0.075-0.293)	45	0.234 (0.003-0.465)	83	0.040 (-0.142-0.222)
Subacute hemorrhage	126	0.503 (0.309-0.697)	45	0.499 (0.213-0.785)	81	0.514 (0.267-0.761)
Hemosiderin	126	0.559 (0.267-0.851)	44	0.788 (0.388-1.19)	82	0.461 (0.096-0.826)
Diffusion restriction	103	0.257 (0.037-0.477)	35	0.539 (0.084-0.994)	68	0.143 (-0.069-0.355)
Venous washout	126	0.394 (0.200-0.588)	43	0.239 (-0.063-0.541)	83	0.495 (0.252-0.738)
Fat	126	0.597 (0.405-0.789)	44	0.879 (0.714-1.04)	82	0.269 (-0.003-0.541)
Contour	128	0.398 (0.261-0.535)	45	0.633 (0.449-0.817)	83	0.226 (0.040-0.412)
Diagnosis	128	0.829 (0.735-0.923)	45	0.816 (0.647-0.985)	83	0.832 (0.7140.950)
Treatment	128	0.580 (0.460-0.700)	45	0.438 (0.213-0.663)	83	0.640 (0.499-0.781)
NA: not applicable, no interobserver variability could be calculated						

Supplementary table 2. Intraobserver reliability

	Overall		Rater 1		Rater 2	
**HBP pattern**	**n**	**Kappa (95%CI)**	n	**Kappa (95%CI)**	**n**	**Kappa (95%CI)**
Extensive part hyper-/isointense	36	0.883 (0.726-1.04)	18	0.753 (0.441-1.06)	18	1.00 (1.00-1.00)
Extensive heterogeneity	36	0.767 (0.553-0.981)	18	0.775 (0.461-1.05)	18	0.753 (0.441-1.06)
Raster	36	1.00 (1.00-1.00)	18	1.00 (1.00-1.00)	18	1.00 (1.00-1.00)
Perilesional hypointense rim	36	0.471 (-0.152-1.09)	18	0.640 (0.001-1.28)	NA	NA
Lesional hyper-/isointense rim	36	0.679 (0.342-1.02)	18	1.00 (1.00-1.00)	18	0.308 (-0.282-0.898)
Atoll Fingerprint	NA	NA	NA	NA	NA	NA
White-bordered flower	36	0.923 (0.774-1.07)	18	0.852 (0.574-1.13)	18	1.00 (1.00-1.00)
Scar	36	0.883 (0.726-1.04)	18	1.00 (1.00-1.00)	18	0.769 (0.475-1.06)
Liquid	36	-0.038 (-0.091-0.015)	18	-0.059 (-0.141-0.023)	NA	NA
Partification	36	0.921 (0.768-1.07)	18	1.00 (1.00-1.00)	18	0.882 (0.659-1.11)
**T2-w pattern**	**n**	**Kappa (95%CI)**	**n**	**Kappa (95%CI)**	**n**	**Kappa (95%CI)**
Extensive heterogeneity	36	0.553 (0.261-0.845)	18	0.649 (0.310-0.988)	18	0.455 (0.014-0.896)
Scar	36	0.828 (0.644-1.01)	18	1.00 (1.00-1.00)	18	0.667 (0.342-0.992)
Liquid	36	0.719 (0.350-1.09)	18	0.640 (0.001-1.28)	18	0.769 (0.342-1.20)
Sinusoidal dilatation	36	0.680 (0.392-0.968)	18	0.769 (0.342-1.20)	18	0.609 (0.213-1.00)
Partification	36	0.721 (0.360-1.08)	18	1.00 (1.00-1.00)	18	0.455 (-0.143-1.05)
**Miscellaneous**	**n**	**Kappa (95%CI)**	**n**	**Kappa (95%CI)**	**n**	**Kappa (95%CI)**
Arterial hypointense rim	36	0.464 (0.066-0.862	18	0.824 (0.493-1.16)	18	-0.091 (-0.232-0.050)
Subacute hemorrhage	36	0.471 (0.112-0.830)	18	0.852 (0.574-1.13)	18	-0.098 (-0.261-0.065)
Hemosiderin	36	0.786 (0.382-1.19)	18	1.00 (1.00-1.00)	18	0.640 (0.001-1.28)
Diffusion restriction	31	0.335 (-0.010-0.680)	15	0.455 (-0.135-1.04)	16	0.213 (-0.039-0.501)
Venous washout	36	0.673 (0.420-0.926)	18	0.852 (0.605-1.10)	18	0.449 (0.053-0.845)
Fat	34	0.522 (0.189-0.855)	17	0.667 (0.304-1.03)	17	0.329 (-0.239-0.897)
Contour	36	0.392 (0.220-0.612)	18	0.507 (0.201-0.813)	18	0.299 (0.023-0.575)
Diagnosis	36	0.942 (0.830-1.05)	18	1.00 (1.00-1.00)	18	0.880 (0.653-1.11)
Treatment	36	0.707 (0.505-0.909)	18	0.903 (0.725-1.08)	18	0.500 (0.167-0.833)
NA: not applicable, no intraobserver variability could be calculated						

Supplementary Table 3. Occurrence of imaging characteristics in HCA versus HCC

Imaging characteristics	Occurrence in HCA (n=48)	Occurrence in HCC (n=14)	Univariable analysis OR (95%CI)	p-value
**HBP pattern**				
Extensive part hyper-/isointense	27 (56)	9 (64)	1.40 (0.408-4.80)	0.592
Extensive heterogeneity	14 (29)	10 (71)	**6.07 (1.63-22.6)**	**0.004**
Raster	19 (40)	1 (7)	**0.117 (0.014-0.973)**	**0.025***
Perilesional hypointense rim	7 (15)	4 (29)	2.34 (0.572-9.59)	0.249*
Lesional hyper-/isointense rim	3 (6.3)	1 (7.1)	1.15 (0.111-12.0)	1.000*
Atoll Fingerprint	7 (15)	0 (0)	0.100 (0.006-1.728)	0.334*
White-bordered flower	2 (4.2)	0 (0)	0.361 (0.019-6.90)	1.000*
Scar	14 (29)	6 (43)	1.82 (0.533-6.22)	0.258*
Liquid	11 (23)	7 (50)	3.36 (0.97-11.7)	0.091*
Partification				**<0.001**
- *No*	44 (92)	3 (21)	**0.025 (0.005-0.127)**	
- *Craquelure*	0 (0)	0 (0)	3.39 (0.066-174)	
- *Focal or mosaic*	3 (6.3)	3 (21.4)	4.09 (0.725-23.1)	
- *Nodule in nodule*	1 (2.1)	9 (57)	62.7 (6.63-592)	
**T2-w pattern**				
Extensive heterogeneity	9 (19)	11 (79)	**15.9 (3.66-69.0)**	**<0.001***
Scar	13 (27)	7 (50)	2.69 (0.790-9.17)	0.120*
Liquid	11 (23)	10 (71)	**8.41 (2.20-32.1)**	**0.003***
Sinusoidal dilatation	23 (48)	0 (0)	**0.019 (0.001-0.321)**	**0.001**
Partification				**<0.001**
- *No*	45 (94)	4 (29)	**0.027 (0.005-0.138)**	
- *Focal or mosaic*	2 (4.2)	2 (14)	3.83 (0.488-30.1)	
- *Nodule in nodule*	1 (2.1)	8 (57)	**62.7 (6.63-592)**	
**Miscellaneous**				
Arterial hypointense rim	13 (27)	1 (7)	0.207 (0.025-1.75)	0.159*
T1-w in-phase hyperintensity	12 (26)[1]	8 (57)	**3.89 (1.12-13.5)**	**0.049***
Hemosiderin	9 (19)[1]	0 (0)	0.074 (0.004-1.28)	0.184*
Diffusion restriction	9	4		**0.001**
- *No*	34 (87)	5 (50)	**0.147 (0.031-0.696)**	
- *Yes*	0 (0)	3 (30)	**55.8 (3.03-1026)**	
- *Equivocal*	5 (13)	2 (20)	1.70 (0.278-10.4)	
Venous washout	1	0		**<0.001**
- *No*	42 (89)	3 (21)	**0.032 (0.007-0.157)**	
- *Yes*	2 (4.3)	8 (57)	**30.0 (5.12-176)**	
- *Pseudo*	2 (4.3)	2 (14)	3.75 (0.478-29.4)	
- *Equivocal*	1 (2.1)	1 (7.1)	3.54 (0.207-60.5)	
Fat	1	0		**0.002**
- *No*	41 (87)	7 (50)	**0.146 (0.038-0.566)**	
- *Less than 50 per cent*	4 (8.5)	7 (50)	**10.8 (2.48-46.5)**	
- *More than 50 per cent*	2 (4.3)	0 (0.0)	0.361 (0.019-6.90)	
Contour				**0.022**
- *Spherical*	22 (46)	1 (7.1)	**0.091 (0.011-0.751)**	
- *Cauliflower*	1 (2.1)	0 (0)	0.663 (0.031-14.2)	
- *Aspecific*	25 (52)	13 (93)	**12.0 (1.45-98.8)**	

HCA and HCC are subgroups of HCA/HCC. The total group of patients with HCA/HCC included 64 patients, but two patients could not be classified as either HCA or HCC (uncertain HCA/HCC).
* Fisher's exact test

Supplementary Table 4. Multivariable analysis of imaging characteristics

Supplementary Table 4A. Dichotomous imaging features

	Pathology diagnosis (n, %)		Univariable analysis OR (95%CI)	p-value	Multivariable analysis OR (95%CI)	p-value
	FNH (n=64)	HCA/HCC (n=64)				
**Suggestive for FNH**						
HBP Extensive part hyper-/isointense	59 (92)	37 (58)	**0.116 (0.041-0.328)**	**<0.001**	0.285 (0.053-1.55)	0.146
HBP White-bordered flower	29 (45)	2 (3.1)	**0.039 (0.009-0.173)**	**<0.001**	0.542 (0.088-3.35)	0.510
HBP Scar	61 (95)	21 (33)	**0.024 (0.007-0.086)**	**<0.001**	NA*	NA*
T2-w Scar	61 (95)	21 (33)	**0.024 (0.007-0.086)**	**<0.001**	**0.012 (0.002-0.071)**	**<0.001**
**Suggestive for HCA/HCC**						
HBP Extensive heterogeneity	9 (14)	25 (39)	**3.92 (1.65-9.31)**	**0.001**	1.57 (0.533-4.65)	0.411
HBP Liquid	4 (6.3)	22 (34)	**7.86 (2.52-24.5)**	**<0.001**	NA*	NA*
T2-w Extensive heterogeneity	8 (13)	22 (34)	**3.67 (1.49-9.04)**	**0.003**	0.448 (0.112-1.79)	0.255
T2-w Liquid	4 (6.3)	22 (34)	**7.86 (2.52-24.5)**	**<0.001**	4.68 (0.700-31.3)	0.111
Arterial hypointense rim	5 (7.8)[1]	14 (22)	**3.25 (1.09-9.65)**	**0.028**	2.71 (0.436-16.9)	0.285

Supplementary Table 4B. Categorical imaging features

	Pathology diagnosis (n, %)		Univariable analysis OR (95%CI)	p-value	Multivariable analysis OR (95%CI)	p-value
	FNH (n=64)	HCA/HCC (n=64)				
HBP Partification	0	0		**0.003**		
- *No*	59 (92)	48 (75)	**0.254 (0.087-0.744)**			
- *Craquelure*	2 (3.1)	0 (0)	0.108 (0.006-2.02) #		NA*	NA*
- *Focal or mosaic*	3 (4.7)	6 (9.4)	2.10 (0.502-8.81)			
- *Nodule in nodule*	0 (0)	10 (16)	**48.6 (2.90-812)** #			
T2 Partification	0	0		**<0.001**		0.124
- *No*	63 (98)	49 (77)	**0.052 (0.007-0.406)**		Reference	
- *Focal or mosaic*	1 (1.6)	5 (7.8)	5.34 (0.606-47.1)		0.196 (0.004-8.51)	
- *Nodule in nodule*	0 (0)	10 (16)	**48.6 (2.90-812)** #		NA#	
Diffusion restriction	12	11		**0.023**		
- *No*	50 (96)	40 (78)	**0.145 (0.030-0.694)**			
- *Yes*	1 (1.9)	3 (5.9)	3.19 (0.320-31.7)		NA[4]	NA[4]
- *Equivocal*	1 (1.9)	8 (16)	**9.49 (1.14-78.9)**			
Venous washout	0	1		**0.001**		0.869
- *No*	60 (94)	45 (71)	**0.167 (0.053-0.527)**		Reference	
- *Yes*	0 (0)	11 (18)	**55.3 (3.32-923)** #		NA#	
- *Pseudo*	4 (6.3)	4 (6.3)	1.02 (0.243-4.26)		0.219 (0.007-7.36)	
- *Equivocal*	0 (0)	3 (4.8)	13.9 (0.773-249) #		NA#	
Fat	1	1		**0.009**		**0.004**
- *No*	55 (87)	49 (78)	0.509 (0.197-1.32)		Reference	
- *Less than 50 per cent*	2 (3.2)	12 (19)	**7.18 (1.54-33.6)**		**23.0 (2.45-216)**	
- *More than 50 per cent*	6 (9.5)	2 (3.2)	0.311 (0.060-1.61)		0.053 (0.001-2.64)	
Contour	0	0		**<0.001**		**0.001**
- *Spherical*	3 (4.7)	24 (38)	**12.2 (3.44-43.2)**		**23.6 (3.86-145)**	
- *Cauliflower*	30 (47)	1 (1.6)	**0.018 (0.002-0.138)**		Reference	
- *Aspecific*	31 (48)	39 (61)	1.66 (0.823-3.35)		0.444 (0.048-4.15)	

NA: not applicable. Missing values are not included in percentages. Missing values are given in grey for categorical imaging features. Odds ratios are calculated for each subcategory of categorical imaging features.
* Not applicable because imaging feature was excluded due to collinearity (correlation coefficient of 0.80 or higher with another imaging feature in Spearman's correlation matrix)
# Odds ratios in univariable analysis obtained by Haldane-Anscombe correction and not calculated in multivariable analysis
$ Excluded due to missing values

Supplementary Table 5. Occurrence of imaging characteristics in B-(I)HCA versus non B-(I)HCA

**Imaging characteristics**	Occurrence in non B-(I)HCA (n=34)	Occurrence in B-(I)HCA (n=14)	**Univariable analysis OR (95%CI)**	**p-value**
**HBP pattern**				
Extensive part hyper-/isointense	14 (41)	13 (93)	**18.6 (2.17-159)**	**0.001**
Extensive heterogeneity	9 (27)	5 (36)	1.54 (0.407-5.85)	0.728*
Raster	18 (53)	1 (7.1)	**0.068 (0.008-0.583)**	**0.003**
Perilesional hypointense rim	2 (5.9)	5 (36)	**8.89 (1.47-53.7)**	**0.017***
Lesional hyper-/isointense rim	2 (5.9)	1 (7.1)	1.23(0.103-14.8)	1.000*
Atoll Fingerprint	3 (8.8)	4 (29)	4.13 (0.787-21.7)	0.171*
White-bordered flower	1 (2.9)	1 (7.1)	2.53 (0.148-43.7)	0.503*
Scar	9 (27)	5 (36)	1.54 (0.407-5.85)	0.728*
Liquid	7 (21)	4 (29)	1.54 (0.370-6.43)	0.708*
Partification				0.803
- *No*	31 (91)	13 (93)	1.26 (0.120-13.2)	
- *Craquelure*	0 (0)	0 (0)	2.40 (0.047-124)	
- *Focal or mosaic*	2 (5.9)	1 (7.1)	1.23 (0.103-14.8)	
- *Nodule in nodule*	1 (2.9)	0 (0)	0.467 (0.022-10.0)	
**T2-w pattern**				
Extensive heterogeneity	6 (18)	3 (21)	1.27 (0.270-6.00)	1.000
Scar	9 (27)	4 (29)	1.11 (0.277-4.45)	1.000*
Liquid	8 (24)	3 (21)	0.886 (0.197-3.98)	1.000*
Sinusoidal dilatation	16 (47)	7 (50)	1.13 (0.324-3.91)	0.853
Partification				0.517
- *No*	31 (91)	14 (100)	5.93 (0.323-109)	
- *Focal or mosaic*	2 (5.9)	0 (0)	0.251 (0.013-4.83)	
- *Nodule in nodule*	1 (2.9)	0 (0)	0.467 (0.022-10.0)	
**Miscellaneous**				
Arterial hypointense rim	11 (32)	2 (14)	0.348 (0.066-1.83)	0.292*
T1-w in-phase hyperintensity	9 (27)	3 (21)	0.727 (0.164-3.22)	1.000*
Hemosiderin	7 (21)	2 (14)	0.619 (0.112-3.44)	0.704*
Diffusion restriction	7	2		0.299*
- *No*	22 (82)	12 (100)	11.6 (0.649-206)	
- *Yes*	0 (0)	0 (0)	2.22 (0.043-115)	
- *Equivocal*	5 (19)	0 (0)	0.086 (0.005-1.54)	
Venous washout	1	0		0.639
- *No*	29 (88)	13 (93)	1.79 (0.182-17.7)	
- *Yes*	2 (6.1)	0 (0)	0.244 (0.013-4.68)	
- *Pseudo*	1 (3.0)	1 (7.1)	2.46 (0.143-42.4)	
- *Equivocal*	1 (3.0)	0 (0)	0.453 (0.021-9.73)	
Fat	1	0		0.803
- *No*	29 (88)	12 (86)	0.828 (0.133-5.14)	
- *Less than 50 per cent*	3 (9.1)	1 (7.1)	0.769 (0.073-8.11)	
- *More than 50 per cent*	1 (3.0)	1 (7.1)	2.46 (0.143-42.4)	
Contour	0	0		0.243
- *Spherical*	15 (44)	7 (50)	1.27 (0.364-4.41)	
- *Cauliflower*	0 (0)	1 (7.1)	12.9 (0.600-278)	
- *Aspecific*	19 (56)	6 (43)	0.592 (0.169-2.08)	

* Fisher's exact test
Molecular analyses were only used in selected patients, therefore presence of B-catenin activation in the group of non B-(I)HCA cannot be excludd.

References

1. (Liver) ACoRCoL-R. LI-RADS Lexicon (terms and definitions) as of June 2021 American College of Radiology. [Available from: https://www.acr.org/-/media/ACR/Files/RADS/LI-RADS/LIRADS-Lexicon-Table.pdf.
2. Bilreiro C, Soler JC, Ayuso JR, Caseiro-Alves F, Ayuso C. Diagnostic value of morphological enhancement patterns in the hepatobiliary phase of gadoxetic acid-enhanced MRI to distinguish focal nodular hyperplasia from hepatocellular adenoma. Radiol Med. 2021;126(11):1379-87.
3. Karam AR, Shankar S, Surapaneni P, Kim YH, Hussain S. Focal nodular hyperplasia: Central scar enhancement pattern using gadoxetate disodium. Journal of Magnetic Resonance Imaging. 2010;32(2):341-4.
4. Cannella R, Furlan A. Mosaic architecture of hepatocellular carcinoma. Abdominal Radiology. 2018;43(7):1847-8.
5. Goshima S, Kanematsu M, Matsuo M, Kondo H, Kato H, Yokoyama R, Hoshi H, Moriyama N. Nodule-in-nodule appearance of hepatocellular carcinomas: comparison of gadolinium-enhanced and ferumoxides-enhanced magnetic resonance imaging. J Magn Reson Imaging. 2004;20(2):250-5.
6. van Aalten SM, Thomeer MG, Terkivatan T, Dwarkasing RS, Verheij J, de Man RA, Ijzermans JN. Hepatocellular adenomas: correlation of MR imaging findings with pathologic subtype classification. Radiology. 2011;261(1):172-81.
7. Bise S, Frulio N, Hocquelet A, Alberti N, Blanc JF, Laurent C, Laumonier H, Balabaud C, Bioulac-Sage P, Trillaud H. New MRI features improve subtype classification of hepatocellular adenoma. Eur Radiol. 2019;29(5):2436-47.
8. Hope TA, Fowler KJ, Sirlin CB, Costa EA, Yee J, Yeh BM, Heiken JP. Hepatobiliary agents and their role in LI-RADS. Abdom Imaging. 2015;40(3):613-25.
9. Chernyak V, Fowler KJ, Kamaya A, Kielar AZ, Elsayes KM, Bashir MR, Kono Y, Do RK, Mitchell DG, Singal AG, Tang A, Sirlin CB. Liver Imaging Reporting and Data System (LI-RADS) Version 2018: Imaging of Hepatocellular Carcinoma in At-Risk Patients. Radiology. 2018;289(3):816-30.
10. Zech CJ, Ba-Ssalamah A, Berg T, Chandarana H, Chau GY, Grazioli L, Kim MJ, Lee JM, Merkle EM, Murakami T, Ricke J, C BS, Song B, Taouli B, Yoshimitsu K, Koh DM. Consensus report from the 8th International Forum for Liver Magnetic Resonance Imaging. Eur Radiol. 2020;30(1):370-82.
